# 3D culturing of human pluripotent stem cells-derived endothelial cells for vascular regeneration

**DOI:** 10.7150/thno.69938

**Published:** 2022-06-06

**Authors:** Edit Gara, Eleonora Zucchelli, Annamária Nemes, Zoltán Jakus, Kitti Ajtay, Éva Kemecsei, Gábor Kiszler, Nikolett Hegedűs, Krisztián Szigeti, Iván Földes, Kristóf Árvai, János Kósa, Kraszimir Kolev, Erzsébet Komorowicz, Parasuraman Padmanabhan, Pál Maurovich-Horvat, Edit Dósa, György Várady, Miklós Pólos, István Hartyánszky, Sian E. Harding, Béla Merkely, Domokos Máthé, Gábor Szabó, Tamás Radovits, Gábor Földes

**Affiliations:** 1 Heart and Vascular Center, Semmelweis University, Budapest, H1122, Hungary.; 2 National Heart and Lung Institute, Imperial College London, W12 0NN, United Kingdom.; 3 Department of Physiology, Semmelweis University, Budapest, H1094, Hungary.; 4 MTA-SE “Lendület” Lymphatic Physiology Research Group of the Hungarian Academy of Sciences and the Semmelweis University, Budapest, H1094, Hungary.; 5 3D HISTECH Ltd., Budapest, H1141, Hungary.; 6 Department of Biophysics and Radiation Biology, Nanobiotechnology & In vivo Imaging Center, Semmelweis University, H1094, Budapest, Hungary and In vivo Imaging Advanced Core Facility, Hungarian Centre of Excellence for Molecular Medicine. www.hcemm.eu, Szeged, Hungary.; 7 Department of Internal Medicine and Oncology, Semmelweis University; PentaCore Laboratory, Budapest, H1083, Hungary.; 8 Department of Biochemistry, Institute of Biochemistry and Molecular Biology, Semmelweis University, Budapest, H1094, Hungary.; 9 Lee Kong Chian School of Medicine, Imperial College - Nanyang Technological University, 636921, Singapore.; 10 Research Centre for Natural Sciences, Budapest, H1117, Hungary.; 11 Experimentelle Herzchirurgie, Ruprecht-Karls Universität, Heidelberg, 69120, Germany.; 12 Department of Cardiac Surgery, University of Halle, Halle (Saale), 06108, Germany.

**Keywords:** human pluripotent stem cells, endothelial cells, angiogenesis tracking, tissue-engineered vascular grafts, multimodality imaging

## Abstract

**Rationale:** Human induced pluripotent stem cell-derived endothelial cells can be candidates for engineering therapeutic vascular grafts.

**Methods:** Here, we studied the role of three-dimensional culture on their characteristics and function both *in vitro* and *in vivo*.

**Results:** We found that differentiated hPSC-EC can re-populate decellularized biomatrices; they remain viable, undergo maturation and arterial/venous specification. Human PSC-EC develop antifibrotic, vasoactive and anti-inflammatory properties during recellularization. *In vivo*, a robust increase in perfusion was detected at the engraftment sites after subcutaneous implantation of an hPSC-EC-laden hydrogel in rats. Histology confirmed survival and formation of capillary-like structures, suggesting the incorporation of hPSC-EC into host microvasculature. In a canine model, hiPSC-EC-seeded onto decellularised vascular segments were functional as aortic grafts. Similarly, we showed the retention and maturation of hiPSC-EC and dynamic remodelling of the vessel wall with good maintenance of vascular patency.

**Conclusions:** A combination of hPSC-EC and biomatrices may be a promising approach to repair ischemic tissues.

## Introduction

Atherosclerosis is one of the most common pathophysiologies of diseases with the highest premature mortality in modern society. Given an ageing population with an increasing prevalence of diabetes mellitus, hyperlipidemia and hypertension, the burden of cardiovascular disease continues to increase globally 1. Peripheral arterial disease is a common manifestation of systemic atherosclerosis, affecting the infrarenal abdominal aorta, iliac, and infrainguinal arteries. Medical interventions, surgical revascularization, and endovascular therapy are the treatment options tailored to keep with individual anatomy and disease characteristics. Surgical strategies include the implantation of vessels or prosthetic grafts 2. However, in small-diameter artery reconstruction, the patency of synthetic grafts is particularly low due to the low flow rate and compliance mismatch between host and graft 3. Due to the challenges in developing polymeric grafts, efforts have been made to generate chemically and biologically modified new materials to improve clinical outcomes 4. However, critical issues related to thrombus formation remain unresolved; therefore, there is a need for novel vascular engineering design of functional, native-like, living conduits with the favorable properties of anti-thrombogenicity, biocompliance, and biomechanical stability. We propose that tissue engineering with decellularised matrices from allogeneic or xenogeneic sources may be a promising approach for treating vascular indications. It remains unclear to what extent exogenous cell delivery can contribute to endothelial regeneration. Earlier reports showed that the fabrication of small-diameter vascular grafts using hiPSC-derived mesenchymal progenitor cells 5 or bilayer of hiPSC-derived endothelial cells and smooth muscle cells (SMC) 6 are feasible *in vitro*. Our preferred strategy is the complete removal of cellular components of the vessel wall to develop a structurally sound three-dimensional (3D) matrix and recellularize them with endothelial cells, differentiated from human pluripotent stem cells (hPSC), such as embryonic stem cells (hESC) and human induced pluripotent stem cells (hiPSC). The main drawback of exogenously administered cells has been their limited survival and rapid clearance from the transplantation site following implantation 7. Adding exogenous cells to decellularised vessels may carry some costs in terms of complexity, regulatory hurdles, and the potential need for immunosuppression but would also provide decisive functional benefits. Here we used a rat model to select and optimize a suitable cell type, and the chosen cell type was further assessed in a canine vascular tissue engineering model. We identified viable and functional vascular cells by *in vivo* molecular imaging of angiogenesis and cell tracking for the small and large animal models. This cellular approach may be promoted as a potential novel means of providing reparative and disease-modifying options for patients with ischemic vascular diseases.

## Results

### Endothelial phenotype and function are improved in 3D culture

**Figure 1:**
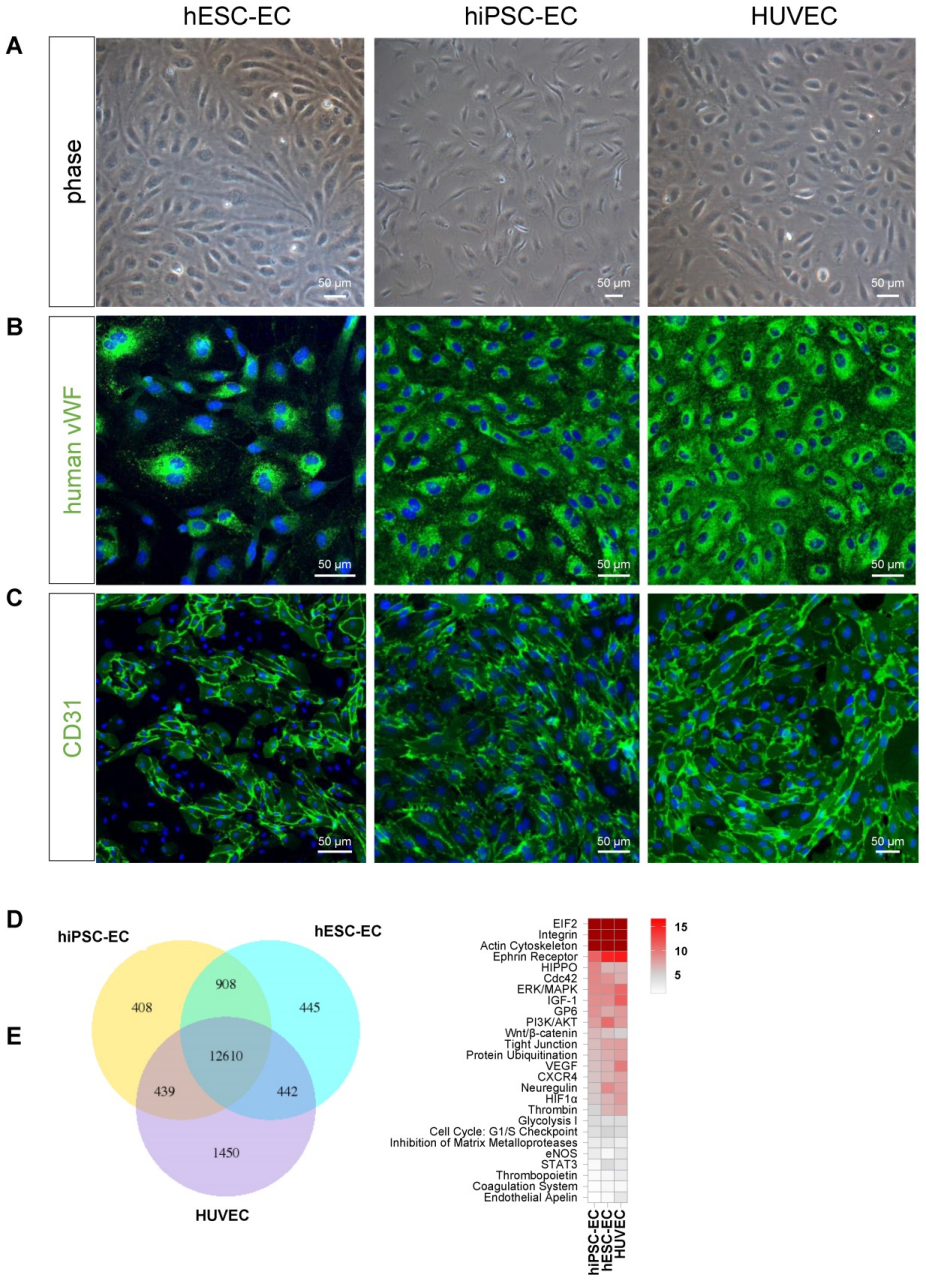
** Differentiation of human embryonic stem cells, human induced pluripotent stem cell resulted in native-like endothelial phenotype.** (**A**) Phase-contrast microscopy images of hESC-EC, hiPSC-EC and HUVEC demonstrating the classical endothelial cobblestone morphology in adherent monolayer culture on collagen. Representative immunofluorescent images for (**B**) von Willebrand factor, vWf and (**C**) CD31 indicated that these endothelial-associated proteins were highly expressed at day 19 of differentiation. Scale bars, 50 µm. (**D**) RNA-seq-based transcriptome comparison between hESC-EC, hiPSC-EC and HUVEC grown on collagen surface. Venn diagram showing the total number in each circle represents the amount of differentially expressed genes between the different comparisons. Only the annotated genes were considered. (**E**) Heat map of shared canonical pathways of the three cell types.

Endothelial differentiation resulted in mature endothelial phenotype *in vitro*. Both differentiated CD31^+^ hESC-EC and hiPSC-EC showed typical endothelial characteristics, such as a cobblestone appearance and stained positive for CD31 and vWF, similarly to those in HUVEC, used as control (Figure 1A-C). RNA sequencing showed a marked similarity in transcriptional profile between hPSC-derived endothelial cells and HUVEC (hESC-EC vs hiPSC-EC: 88%; hESC-EC vs HUVEC: 80%; hiPSC-EC vs HUVEC: 80%, Figure 1D). Common pathways (75% of all genes) comprise networks for arterial-venous specification, angiogenesis, cellular junctions and structure (Figure 1E). Despite expressing endothelial markers CD31 and VE-cadherin at high levels, some of the hESC-EC and hiPSC-EC underwent endothelial-mesenchymal transition (EndoMT) and transdifferentiated into other vascular cell types as FSP1^+^ smooth muscle cells (Figure S1A). TGFβ-2, or ALK5 inhibitor SB431542 did not affect EndoMT-related gene expression or the distribution of CD31^+^/FSP1^+^ cell populations (Figure S1BCD).

### Implantation of human PSC-EC-laden hydrogel increases angiogenesis and perfusion *in vivo*

**Figure 2:**
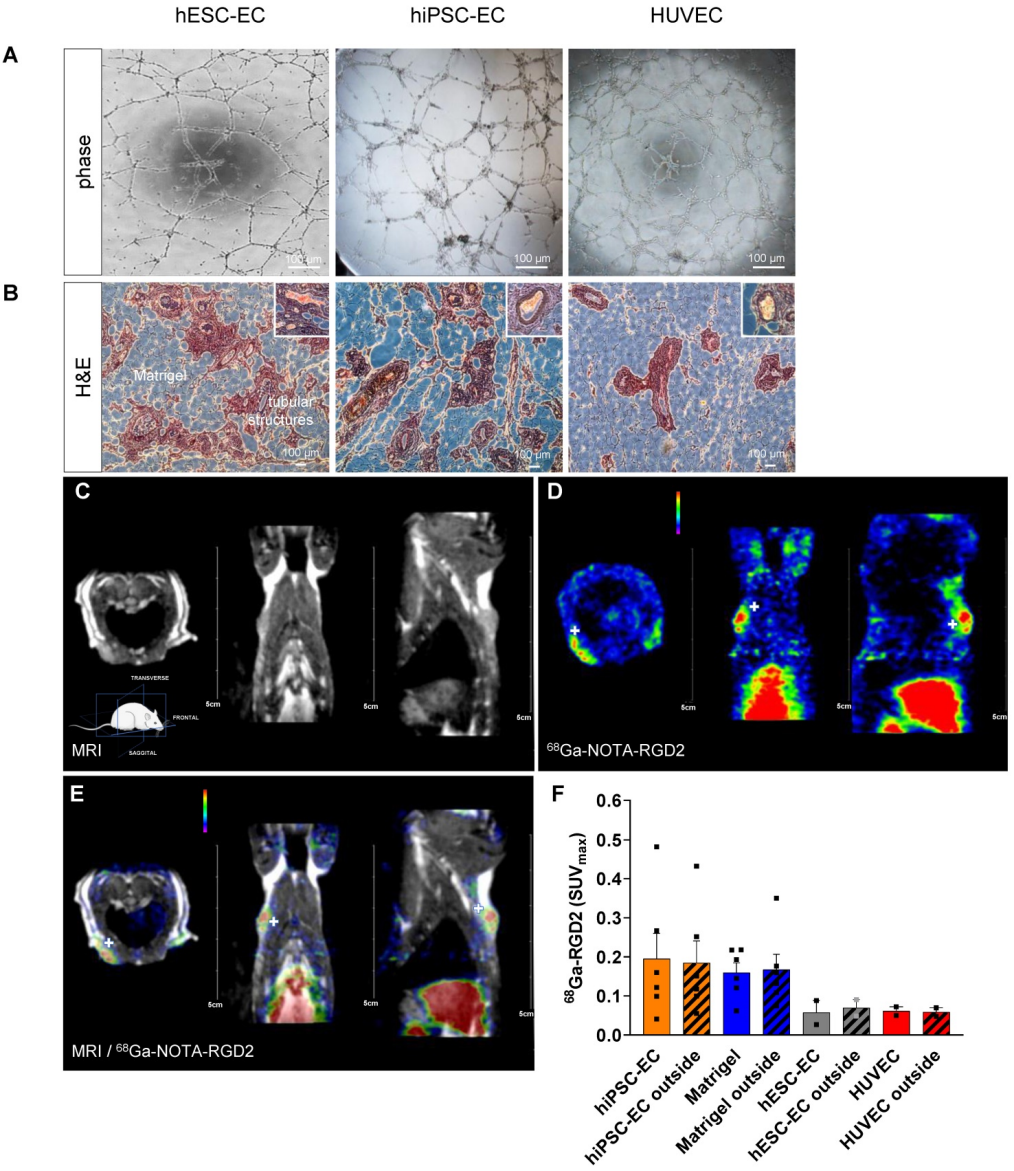
** Functional endothelial cell-laden hydrogel implants.** (**A**) Representative images showing tube formation angiogenic activity by capillary structures of hESC-EC, hiPSC-EC and HUVAC on growth factor-reduced Matrigel. (**B**) Hematoxylin-eosin staining of subcutaneous implants of hESC-EC, hiPSC-EC or HUVAC in Matrigel hydrogel in nude rats 14 days after subcutaneous implantation are shown. Scale bars, 100 µm. Presence of red blood cells in tubular structures suggests a functional link to recipient circulation, inset panels. See also Video S1 and S2 on 3DHisTech analysis of 3D vascular structures. (**C**) Magnetic resonance imaging (MRI, co-registration with (**D**) positron emission tomography (PET, showing (**E**) ^68^Ga-NOTA-RDG2 uptake in the endothelial cell-implanted animals. PET and PET/MRI images feature white crosshairs at the plug implantation and increased vascularization sites. MRI image panel is shown without the same for ease of anatomical assessment. (**F**) [^68^Ga]-NOTA-RDG2 expressed as maximum standardized uptake values (SUV_max_) in donor hydrogel (solid bar) and surrounding recipient tissue are also measured (stripped bar), mean ± SEM, n = 4-6, Kruskal-Wallis nonparametric test.

**Figure 3:**
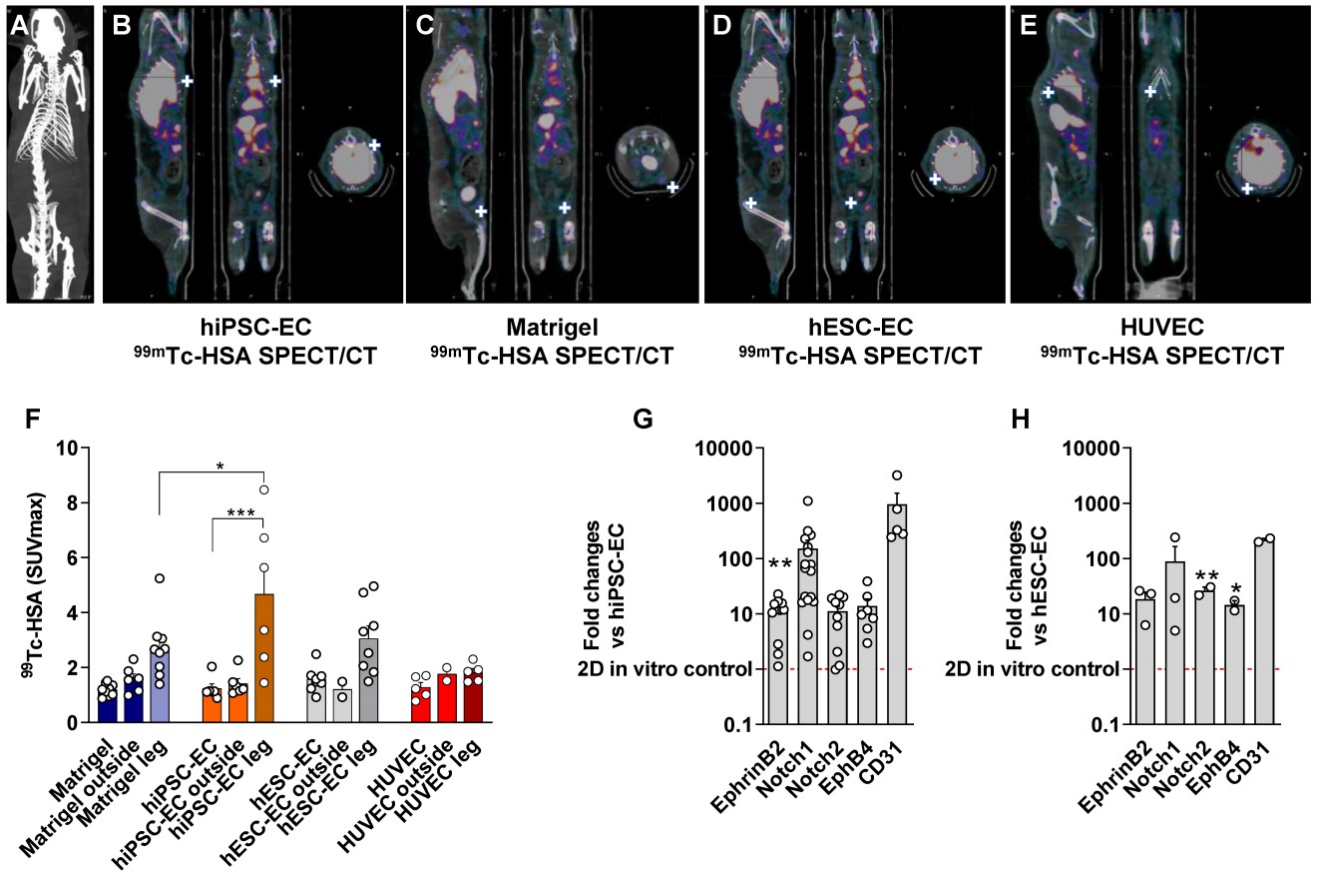
***In vivo* multimodality imaging shows increased perfusion in response to hPSC-EC.** Comparison of hydrogel-based endothelial plugs on ^98m^Tc-human serum albumin (HAS) to assess perfusion of newly formed vessels. (**A**) Representative CT scan to show whole animal. (**B**) Multimodality quantitative SPECT/CT imaging revealed increased perfusion after hiPSC-EC, (**C**) human cell-free Matrigel, (**D**) hESC-EC and (**E**) HUVEC plugs. White crosshairs showing the site of implants. (**F**) Bar graph showing SUV_max_ values were calculated, mean ± SEM. (**G-H**) Grouped bar diagrams show expression of arterial (ephrin B2, Notch1, Notch2), venous (EphB4) and common (CD31) endothelial marker genes at two weeks after subcutaneous transplantation of hESC-EC (**G**) and hiPSC-EC (**H**) in athymic rats. mRNA levels are normalized to those in pre-implanted control cells, n = 6. * *P* < 0.05, ** *P* < 0.01, *** *P* < 0.001, one-way ANOVA. See also Figure S2.

Differentiated CD31^+^ endothelial cells from hESC line H7 and hiPSC line IMR90 showed comparable vascular network formation activity to those in native HUVEC when plated on Matrigel (Figure 2A). To test angiogenic activity *in vivo*, we mixed hESC-EC or hiPSC-EC with Matrigel and vascular growth factors and injected them subcutaneously into athymic rats. No xenogeneic cellular implant-related adverse effects or inflammatory reactions were observed in cellular implants and host animals. Like *in vitro* angiogenic activity, hematoxylin-eosin staining of explanted plugs showed capillary-like structures within and around the plug at two-weeks follow up. The presence of red blood cells confirmed functional coupling between host and recipient vessels in the lumen in *ex vivo* histology (Figure 2B, inset panels; Videos S1). As shown by immunohistochemistry data from digital 3DHistech scans, the hESC-EC and hiPSC-EC formed capillary-like structures *in vivo*, with an average of 20.8±0.9 % vascular coverage of the total plug volumes. For simultaneous positron emission tomography (PET)/magnetic resonance imaging (MRI) imaging (Figure 2C), we labelled α_v_β_3_ integrin affine agent NOTA-RGD2 with gallium (^68^Ga). We used this for α_v_β_3_ integrin biodistribution two weeks after implantation (Figure 2DE). After intravenous administration, ^68^Ga-NOTA-RGD selectively bound to free α_v_β_3_ integrin on the cell membrane via the cyclic RGD motif, and detected new vessel formation marker of vascular endothelial cell migration and invasion 8. Axillary and inguinal administration of hiPSC-EC, and cell-free Matrigel plugs with growth factors (used as sham reflecting recipient rat cell-induced changes in angiogenesis and perfusion), showed the highest level of radioactive labelling inside the plugs (SUV_max_: 0.2 ± 0.07 and 0.16 ± 0.03, respectively) and similarly in the surrounding area (Figure 2F). Plugs with hESC-EC and control HUVEC both showed modest upregulation in the ^68^Ga-NOTA-RGD activity. Direct comparative analysis of the area inside the implants (solid bars) versus the surrounding compartments within the host tissue (patterned bars, “outside”) showed that revascularization and new coupling between donor and host vasculature formed parallelly (P = 0.9). The perfusion of implanted constructs and surrounding host tissue was quantitated by single-photon emission computed tomography (SPECT)/computer tomography (CT). We found an increased perfusion activity in the hydrogel-based plugs by assessing the biodistribution of ^99m^Tc-labelled albumin injected intravenously via the tail vein. Both hESC-EC (SUV_max_: 1.6 ± 0.2) and hiPSC-EC (SUV_max_: 1.2 ± 0.2) increased perfusion, comparable to those in control HUVEC (SUV_max_: 1.3 ± 0.2) (Figure 3A-E and Figure S2). Perfusion in human cell-free Matrigel was also increased both within the plug and surrounding tissue. After plug transplantation into the axillary or inguinal areas,^ 99m^Tc-labelled albumin accumulation in injected limbs was significantly increased for all cell types. As the maximum standardized uptake values (SUV_max_) in injected limbs were found after the hiPSC-EC implantations (Figure 3F), we have chosen to use this cell type as a further scaffold-based peripheral artery model. After two weeks of engraftment, we observed a Notch1-mediated upregulation of human endothelial genes both in hESC-EC and hiPSC-EC (P < 0.01), compared to those in 2D culture before transplantation *in vitro* (Figure 3GH).

### Human iPSC-EC-seeded decellularised aortic segments are functional as grafts

**Figure 4:**
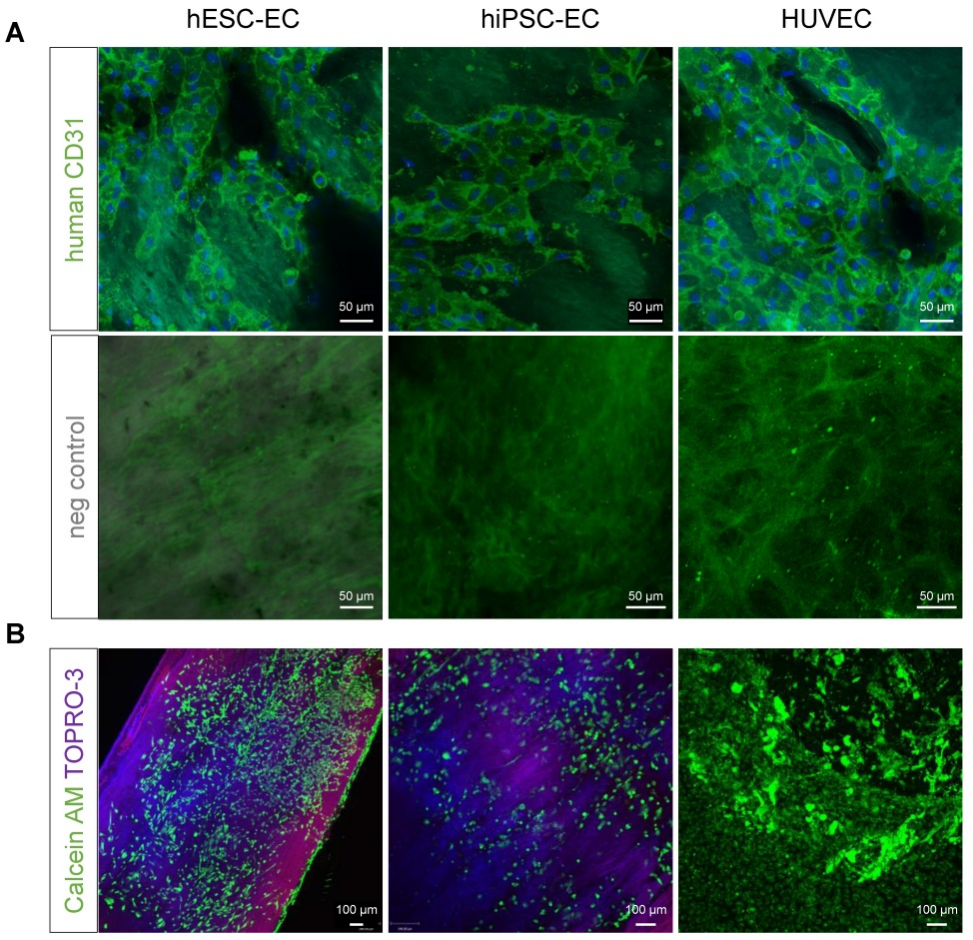
(**A**) Previously decellularised vessel walls were recellularised with either hESC-EC, hiPSC-EC or HUVEC and stained with anti-human CD31 antibody (BDBiosciences, green). Hoeschst for nuclear staining and respective negative controls are also shown. Nuclei are counterstained with Hoechst (blue). (**B**) Live cells stained with vital dyes calcein AM (green, excitation at 495 and emission at 515 nm) and necrosis marker TO-PRO3 (excitation at 642 nm and emission at 661 nm). Scale bars, 50 µm (**A**) and 100 µm (**B**).

**Figure 5:**
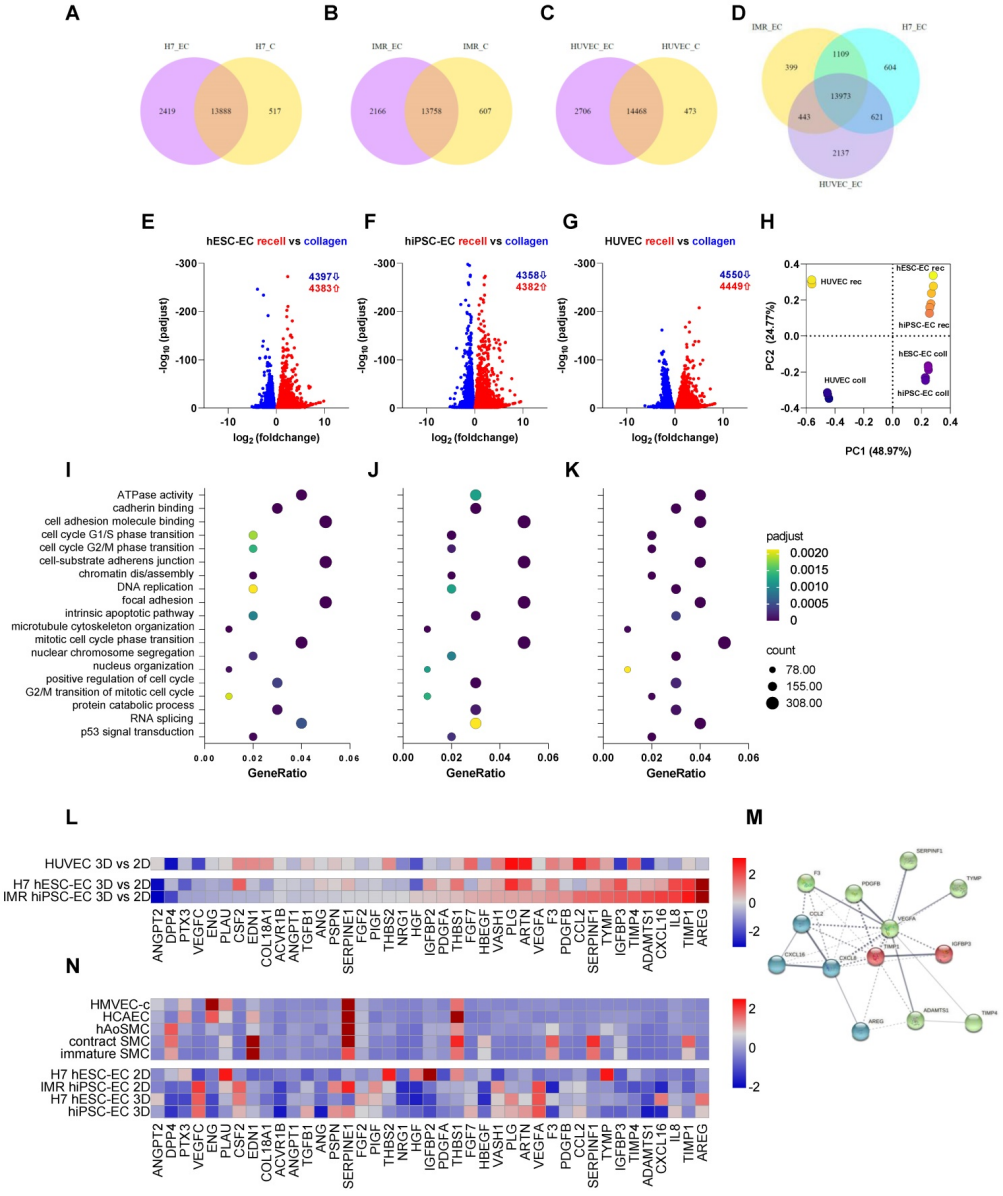
** RNAseq-based transcriptomics profiling of endothelial cells cultured either on collagen or decellularised vascular matrix.** (**A-D**) Venn diagrams showing the differential expression profile of H7 hESC-EC, IMR hiPSC-EC and HUVEC cultured on collagen vs. decellularised vascular wall. (**E-G**) Volcano plots and (**H**) principal component analysis show differential expressions of genes in the three cell types. The first two principal components of the gene expression dataset are plotted here for each of the samples. (**I-K**) Enriched GO terms shows as dot plots. The 19 GO processes with the largest gene ratios are plotted in order of gene ratio. The size of the dots represents the number of genes in the significant differentially expressed gene list associated with the GO term and the colour of the dots represent the P-adjusted values. (**L**) RNA-seq-based heat map showing angiogenesis and cell-matrix adhesion protein profiling in 3D vs 2D endothelial cultures. (**M**) Functional association network diagram EC are generated by String DB pathway analysis. n = 3 biological replicates. (**N**) Angiogenesis and cell-matrix adhesion protein profiling in 2D and 3D endothelial cultures as well as native aortic and hiPSC-derived smooth muscle cells (SMC). Contractile and synthetic SMC are both shown. Further controls used are human microvascular endothelial cells (HMVEC) and human coronary artery endothelial cells (HCAEC-c). Heat map shows expression of angiogenesis-related proteins of hESC-EC and hiPSC-EC (2D and 3D cultures, respectively). Proteomics in hESC-EC and hiPSC-EC are normalised as z scores. n = 2 biological replicates, 4 technical replicates. Array membranes were visualized by chemiluminescent detection; pixel densities were quantified by ImageJ software. Protein quantities were equalized in each experimental setting; passages between 3 and 5 were used.

CD31^+^ hiPSC-EC, hESC-EC and HUVEC remain viable after reseeding on acellular human aortic matrices, as 100 % of cells were positive for cell-permeant vital probe calcein AM, without detectable levels of necrosis marker TO-PRO3 (Figure 4A-B). Matrisome-based comparison showed that decellularised vessel matrix was composed of extracellular matrix proteins overlapping with those found in Matrigel (Figure S3A), in the vascular vessel wall and proteins involved in platelet regulation and collagen-deposition (Figure S3B). RNA-seq analysis of hESC-EC, hiPSC-EC and HUVEC grown either on collagen or decellularised vessel was performed (Figure 5). Head-to-head comparison of the three cell types suggested a similar activation profile in 3D culture *in vitro* (Figure 5A-G). This was also found for non-coding RNAs (Figures S4 and S5). The principal component analysis also supported a significant overlap in gene networks during recellularisation (Figure 5H and Figure S4). Pathway network analysis identified key changes in the cell cycle program and cell adhesion and junctions (Figure 5J-K). RNA-seq and proteome profiling revealed abundant secretion of selected proteins in 2D cultures of hESC-EC and hiPSC-EC. The 3D culture-induced key angiogenesis-related protein secretions (Figure 5L-N) were in keeping with the maturation of endothelial phenotype in both hPSC cell types. Key angiogenesis-related proteins were expressed, such as Activin receptor 1B, CCL3, CXCL16, IL-8, PDGFB, TGFB1, vasohibin and VEGF-A. Gene network analyses suggested that secreted proteins such as VEGF, angiopoietin-1, TIMP-1, and Serpine1 are key members of several angiogenesis and cell-matrix adhesion networks (Figure 5M). These were different to those in hiPSC-derived and native smooth muscle cells used as controls (Figure 5N).

Factors involved in regulating platelet aggregation or fibrinolytic pathways, such as TIMP4 and plasminogen, were also activated in hESC-EC and hiPSC-EC in 3D cultures. To address this, we tested how extracellular matrices and biomechanical cues affect the function of hPSC-EC. Platelet and fibrin(ogen) deposition from flowing heparinized blood onto the decellularized, reseeded vessel walls was tested in a parallel-plate flow chamber model *in vitro*. Reseeding the vessel wall, especially with hiPSC-EC, decreased fibrin(ogen) deposition on the intima layer (P < 0.001), restored low platelet coverage over the media layer, and did not perturb the hemostatic capability of the outermost layer (Figure S6AB). In the isolated vessel assay 9, we have shown that hPSC-EC have an acute vasoactive effect on isolated aortic rings. We directly assessed the vasoactive effects of hESC-EC, hiPSC-EC, and control HUVEC (Figure S7). The rat aortic rings had an increased diameter in response to the conditioned medium of hiPSC-EC (P = 0.009). A vasodilating trend (adjusted P = 0.09) was also observed on the hESC-EC-conditioned medium. The conditioned endothelial medium did not affect acetylcholine-induced vasodilation, suggesting that hESC-EC and hiPSC-EC secretome does not act on cholinergic receptors but distinct pathways such as the arachidonic acid signaling 10.

### Remodelling in large animal model

**Figure 6:**
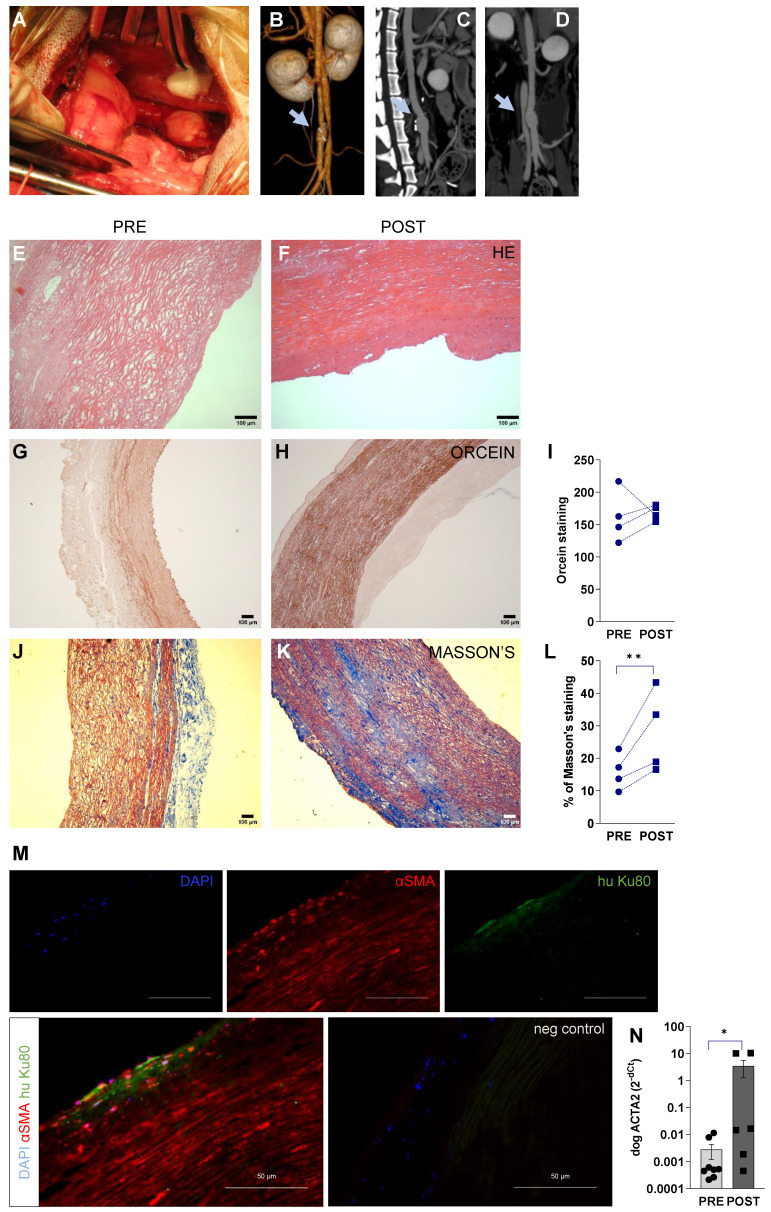
** Surgical procedure and imaging of recellularised aortic graft implantation in canine model.** (**A**) hiPSC-EC graft implantation into the peripheral artery by performing end-to-end anastomosis in dog infrarenal aorta, n = 4. (**B-D**) 3D and multiplanar reconstruction CT images of the implanted hiPSC-EC graft. Grey arrows showing the site of the graft. (**E-F**) Hematoxylin-eosin, (**G-I**) orcein and (**J-L**) Masson's staining before surgery (PRE) and at one-week follow-up (POST) **P < 0.01, paired Student's t-test. Immunohistochemistry of α-smooth muscle actin (red), human nuclei (green) and nucleus label DAPI (blue) in the luminal segment of the aortic wall at day 7 after surgery (M). Scatter-plot and bar graphs show mRNA levels of canine ACTA2 at implantation and at day 7 after surgery (**N**). Normalized mRNA levels are shown as 2-ΔΔCt, mean ± SEM, *P < 0.05, Wilcoxon matched-pairs signed-rank test, from n = 4 animals. y-axis, note log scale.

In our large animal model of abdominal aortic replacement, we decellularised aortic grafts, recellularised with hiPSC-EC, and implanted them into beagle dogs using aortic anastomosis in the infrarenal segment (Figure 6A). The primary endpoints were feasibility and safety, including the absence of infection, aneurysm, and mechanical graft failure as assessed by patency at one week. This model used standard perioperative anticoagulation, antiplatelet protocols, and clinical-grade functional/morphological imaging. The residual lumen diameter was assessed by perioperative CT angiography (Figure 6BCD) and flow was obtained at mid-graft, and proximal and distal anastomoses by serial duplex ultrasound (Figure S8). Grafts remained patent, and the mean luminal diameter was 6.75 ± 0.48 mm both at the proximal and distal segments, and mid-region dilatation was 1.25 ± 1.00 mm compared to anastomoses. No hematoma or inflammatory fluid collection was observed around the grafts (Figure S8A). Inside the grafts, no thrombus formation was seen; minimal intimal thickening was visualized at the anastomotic sites (intima-media thickness at the proximal anastomosis, 1.37 ± 0.10 mm; intima-media thickness at the distal anastomosis, 1.35 ± 0.13 mm). No significant change in flow velocity was observed (peak systolic velocity, P = 0.2; end-diastolic velocity, P = 0.45, two-way ANOVA) in the distal and proximal anastomoses and the mid-regional area of the grafts (Figure S8B). The triphasic flow was measured at each segment of the abdominal aorta. Next, we assessed the histological and biochemical properties of the recellularised aortic graft. We have found that it was resistant to physiological load during implantation with stable fabrication consistency. Our histology data have shown that damaged or lack of endothelium can disrupt the physiological balance of the vessel, leading to accelerated smooth muscle proliferation 11. This phenomenon was detectable by the progressive remodeling and thickening of the media layer of the recellularised vessel. Here we found that this process was driven by the repopulation of lamina media by smooth muscle cells, resulting in extracellular matrix production and remodeling of the vascular structure. This confirms data from previous studies where host smooth muscle cells were found to migrate and produce extracellular matrix due to endothelial dysfunction 12. The decellularised media of vascular wall showed a remodeling (Figure 6EF), accumulation of orcein-positive elastic fibers (Figure 6G-I), and Masson's positive collagen (Figure J-L) at one week after transplantation. Remodeling was also confirmed in decellularised grafts during 6-months follow-up (Figure S9). We found that the majority of αSMA-positive cells were endogenous canine cells (≥ 95%, Figure 6M) in media (and some cells in intimal) layer after remodeling. mRNA levels of canine ACTA2 (αSMA) were also increased during repopulation (P = 0.03, Figure 6N). The number of human iPSC-derived cells was below 1% in the regenerated media layer at day 7, suggesting that exogenous delivery of hPSC may not contribute significantly to media regeneration. By confocal Cellvizio endoscopy, we have provided a detailed 3D fiber alignment in the intimal layer. Grafts demonstrated no significant differences in textural features of extracellular matrix filaments (like collagen) in the intimal layer between surgery and 7 days post-surgery images (Figure S10A-D).

### Endogenous vascular repair versus exogenous cell-initiated re-endothelization

**Figure 7:**
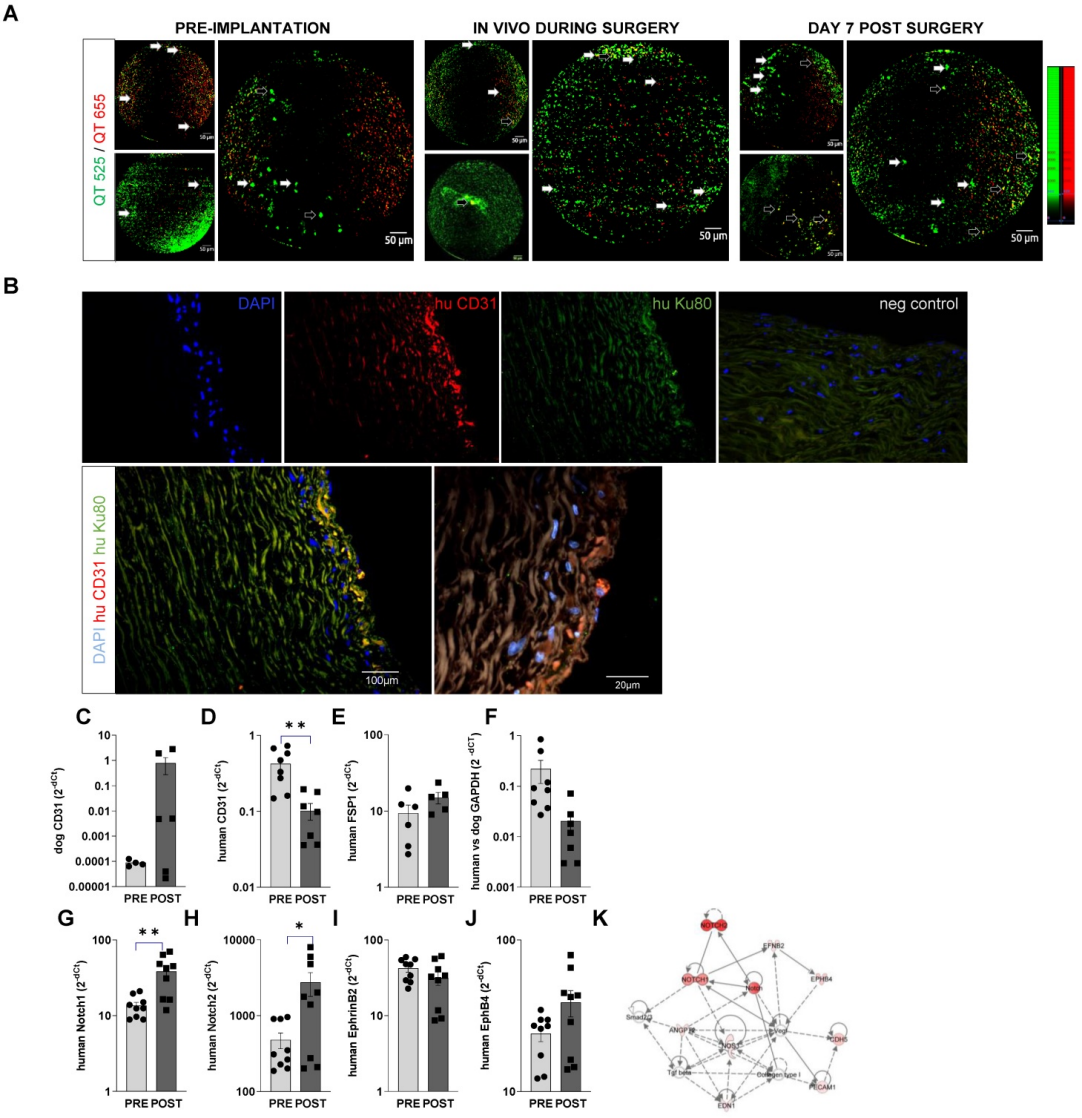
***In vivo* retention of hIPSC-EC on recellularized vessel wall.** (**A**) Confocal fiberoptic endo-microscopy imaging to track fluorescently labelled cells seeded onto luminal surface of the decellularised vessel wall before, during, and 1 week after surgery. QTracker (QT) 525 and -655 vital dyes are used to improve cell signal to background and non-specific autofluorescence ratio. Resolution is 3 µm, with 8 Hz sampling rate. The field-of-view is 800 µm in diameter. Empty arrows point to hiPSC-EC with fluorescent signal in both channels, white arrows to cells that accumulated QT515 (green) only. (**B**) Human iPSC-EC were costained for human nuclei Ku80 (green) and anti-human CD31 (red) in the luminal segment of the aortic wall after recellularization by immunohistochemistry. Nuclei were counterstained with DAPI (blue). Scatter-plot and bar graphs show mRNA levels of (**C**) dog CD31 (**D**) human CD31, (E) human mesenchymal marker FSP1, (**F**) human versus dog GAPDH and (**G-J**) endothelial specification (Notch1, 2, ephrin B2, EphB4, at implantation and at day 7 after surgery. mRNA levels are shown as 2-dCt, mean ± SEM, n = 5-6 samples, from 4 animals. *P < 0.05, **P < 0.01, Student's t-test. (**K**) Ingenuity Pathway analysis of the genes tested.

Cellvizio endomicroscopy showed that seeded hiPSC-EC labelled with vital fluorescent trackers are retained on the inner surface of the recellularized graft during operation and at 1 week after surgery (Figure 7A). Cells adhered efficiently to the surface of the scaffolds, without any significant deep penetration into the matrix, as also shown in cross-sectional histology images (Figure S10E-G). Staining with antibodies specific for human CD31 and human nucleus Ku80 suggested the retention of hiPSC-EC on the aortic wall. In combination with canine/human-specific real-time PCR assays, we further confirmed the retention of endothelial cells on the vessel wall. We found an increasing trend in canine-origin mRNA, a decrease in human CD31 and GAPDH mRNA levels and no increase in human FSP1 mRNA levels (Figure 7C-F). This suggests that more canine endothelial cells are involved in reendothelization than those from exogenously implanted hiPSC-EC over the one-week follow-up. This is in line with recent findings 13, 14 showing that most new cells come from the actively proliferating endogenous local subpopulation during vascular regeneration. Similar to *in vitro* data and the small animal experiments, *in vivo* conditioning of hiPSC-EC seeded on decellularised aorta induced upregulation of arterial Notch 1 and 2 mRNA levels at a one-week time point (Figure 7GH). This suggests that *in vivo* niche may maintain endothelial phenotype (Figure 7I-K).

## Discussion

Most of the cardiovascular cell therapy trials have ended with moderate or non-beneficial outcomes so far. Whilst feasibility and safety results are acceptable, inconsistent functional benefits and mortality outcomes of these trials limit their clinical application in cardiovascular disease. Borderline functional effects may be due to fast clearance of the exogenously administered cells from the transplantation site, regardless of the administration route. Furthermore, the limited survival rate hampers the activity of the retained cells. To overcome these issues, we need to address improved integration and functional activity of the donor cells. Different delivery methods and vehicles have been proposed to increase cell retention and viability after engraftment. Human pluripotent stem cells-derived endothelial cells may be ideal for therapeutic neovascularization of ischemic tissues; these can meet therapeutic expectations only if they possess sufficient functional activity to support the physiological mechanisms of the vascular system. Human PSC-EC is readily available as undifferentiated hPSC and can be efficiently differentiated towards endothelial lineage using established protocols 15-17. Intravenously administered iPSC-derived Flk1^+^ cells were shown to be recruited to the site of vascular injury, enhancing reendothelialization and suppressing neointimal hyperplasia 18.

This study showed that endothelial derivatives of hPSCs develop mature endothelial characteristics and functional properties in 3D cultures. Using decellularized human vascular scaffolds, we demonstrated that hESC-EC and hiPSC-EC recellularized the matrix, remain viable, and undergo further maturation *in vitro* and *in vivo*. Levels of proteins related to cell-matrix adhesion, collagen XVIII, MMP8, MMP9, TIMP1, MCP-1, showed a robust increase in 3D culture, thereby increasing the adhesive capacity of cells upon the reseeding of matrices. Human ESC-EC and hiPSC-EC also expressed angiogenesis-related factors. The importance of the proteome in triggering angiogenesis is well described; however, the novelty of this finding suggests that cell-free therapeutic use of the secretome of hESC-EC and hiPSC-EC may be enhanced via 3D culture preconditioning. The efficacy of cellular engraftment was low in previous clinical trials, and a regenerative secretome may be the desired treatment approach 19. Having proven that the receptor for VEGF and VEGFR2/KDR is highly expressed in hESC-EC and hiPSC-EC, we propose that the secretome has a strong pro-angiogenic potential compared to adult endothelial cells. VEGF, Tie-2 and Notch1 signaling trigger tip cells to initiate new arterial branches during sprouting angiogenesis 20, 21. Direct regulation of myogenic tone by hESC-EC and hiPSC-EC is also important, considering the development of vascular grafts for clinical use. By using aortic rings as an *in vitro* isolated vessel platform, we have shown that acute exposure of vascular surfaces to hPSC-EC supernatant has a direct vasoactive effect. Factors involved in antiplatelet and fibrinolytic pathways, such as TIMP4 and plasminogen, were activated in both hESC-EC and hiPSC-EC cell types in 3D cultures, as illustrated by proteome profiling. Similar to native endothelial cells, hESC-EC and hiPSC-EC reduced platelet activation in 3D conditions, as shown by decreased platelet-derived Rantes (CCL5) levels in platelet-rich plasma. A previous study has also demonstrated that hESC and hiPSC derived endothelial cells have similar antiplatelet effects on a poly-caprolactone scaffold 22.

Recent approaches have been based on natural matrices to overcome the limitation of autologous grafts, which has led to the commercialization of decellularized products. To improve the properties of these grafts, autologous endothelial cells and smooth muscle cells from different origins were seeded onto decellularized matrices and then tested in ovine 23 and canine models 24. Here we showed the *in vivo* delivery, retention and survival of hiPSC-EC on scaffold-based vascular grafts in a large animal model. For reendothelization of damaged or disrupted (or decellularised, in our project) vascular surfaces, hESC-EC/hiPSC-EC, along with endogenous canine endothelial cells, formed a single endothelial cell layer to replace intima on solid decellularised vascular surfaces of mid-size arteries. This endothelial recovery consists of three main processes, i.e. migration, adhesion and proliferation. This process was well documented by the detailed RNAseq analysis. We could track labelled cells on the aortic surface by taking advantage of high resolution intravital confocal imaging. Cells used for preseeding the cell-free vascular grafts may help colonize injured/cell-free luminal surfaces. We found that cells retained on the luminal surface under high pressure and high wall shear stress were viable during the follow-up.

Our other *in vivo* approach was to preseed scaffold with cells *ex vivo* and inject cells suspended within a hydrogel made of extracellular matrix Matrigel and vascular growth factors into immunocompromised rats. We used high-sensitivity multimodality molecular imaging combined with angiogenesis-specific radioactive or fluorescent labelling to visualize the cellular fate of implanted hPSC-EC and track angiogenic processes vitro and *in vivo*. Fluorescent histology is of a further advantage as most cell therapy protocols need to determine engraftment of the transplanted cells. Reconstructed SPECT/CT images revealed an increase of perfusion in the areas of transplanted endothelial cells in axillary and inguinal injection sites, and a parallel increase in perfusion was also measured in the limbs. This suggests a relevant angiogenic activity of endothelial cells, resulting in the formation of new vessels during transplantation and increased flow even in healthy limbs. The degree of perfusion increase was similar in the case of implanting hPSC-EC and HUVEC. This indicates that hESC-EC and hiPSC-EC are stable endothelial cell cultures with the plasticity to preferentially form specific, branched vascular architectures. Injection of hydrogel without cells induced local perfusion, which may be a consequence of angiogenic growth factor released from the gel. We also showed that the hydrogel-based construct could shield cells from mechanical stress observed during subcutaneous injection and allowed rapid gelation to entrap the cells and prevent their clearance from the injection site. Activation of RGD peptide-dependent signal in PET/MRI scans preceded soluble human serum albumin (HSA)-related signals in SPECT/CT scans. An increase in ^99m^Tc-HSA accumulation reflects completed angiogenesis, and consequent increase in perfusion capacity of the implant. An increase in RGD but not HSA was representative for early angiogenesis without a completed link between the donor and recipient vascular structures. Head-to-head comparison of donor construct and surrounding areas suggests improved vascularization of the tissue by vascular/perivascular cell recruitment may improve donor cell engraftment. Here we described an approach for tracking cell retention and quantifying the angiogenic and reendothelization activity of hPSC-EC *in vivo*. To assess the potential clinical benefits of our revascularization approach, reliable cell tracking is required. This should focus on cell survival rate, proliferation, maturation, migration, and angiogenic activities of the transplanted cells 25-27.

In summary, our findings from *in vitro*, small animal and preliminary large animal data suggest that exogenous administration of hPSC-EC can be used for vascular regeneration. This preclinical study may substantiate hPSC-EC-biomatrix engineering potential in vascular therapy and promote further clinical approaches for vascular reconstruction.

## Methods

### Human pluripotent stem cell culture and endothelial differentiation

Endothelial cells were derived from human embryonic stem cell WA007 line (H7) and human induced pluripotent stem cell line IMR-90-4 (both from WiCell Bank, Madison, Wisconsin, USA). Pluripotent stem cell colonies were maintained in mTeSR1 medium (Thermo Fisher Scientific, Waltham, Massachusetts, USA) in feeder-free conditions on Matrigel (BD Biosciences, Wokingham, UK) coated plates. For endothelial differentiation, human pluripotent stem cell colonies were enzymatically dissociated and were cultured in suspension in low-attachment six-well plates (Corning, NY, USA) to develop embryoid bodies (EB). EBs were maintained in endothelial growth medium-2 (EGM2) (Lonza, Basel, Switzerland) for 4 days. At day 5 of differentiation EBs were seeded on gelatin-coated flasks. At day 13 of differentiation CD31^+^ endothelial cells (hESC-EC and hiPSC-EC) were sorted by fluorescence cell sorter (BD FACSAria; BD Biosciences) and expanded in EGM2 on 0.1% gelatin (Sigma-Aldrich, St. Louis, Missouri, USA). Endothelial cells at passage 2-5 were used for experiments. Human umbilical vein endothelial cells (HUVEC, Lonza) were used as control endothelial cells; these were cultured and maintained as hESC-EC and hiPSC-EC.

### Matrigel tube formation

Tube formation assay was performed on endothelial cells as described previously 15.

### Endothelial cells and smooth muscle cells for proteome profiling

Angiogenesis proteome profiling was carried out with Proteome Profiler Human Angiogenesis Array Kit (RnD Systems, ARY007). The human soluble receptor proteome profiling array was performed with Human Soluble Receptor Array Kit Hematopoietic Panel (RnD Systems, ARY011). The sample preparation and experimental setup followed the product catalogue guide. In addition to endothelial cells (hPSC-EC and HCAEC microvascular cells), human smooth muscle cells have also been used as controls. Human induced pluripotent stem cells (IMR90-4) were cultured in a 2D cell culture system in 24 well-plates and under standard culture conditions (37^o^C, 5% CO2). The starting cell number was 10^5^ cells/cm^2^; we expanded cells in stem cell medium for two days. For the differentiation stage, glucose-based RPMI1640 medium was used in the presence of VEGF-A, FGF2 and B27-supplement. After starting the differentiation, contractile subgroup of smooth muscle cells (SMC), human PDGF-BB and TGFß proteins were added to the basal medium. Human aortic SMC (Lonza) was used as further smooth muscle cell control. Pixel densities of the x-ray films were analysed by ImageJ software. Protein-protein interaction networks functional enrichment analysis was performed by String DB (string-db.org).

### Antiplatelet assay

To characterize platelet adhesion and fibrinogen levels on vessel layers of constructs reseeded with hESC-EC, hiPSC-EC and HUVEC, we used a previously described flow-chamber model to test the vessel samples thrombogenic surfaces 28, 29. Frozen cross-sections (10 µm) of homograft samples placed on poly-L-Lys-coated slides were perfused at 0.5 mL/min flow rate in a 0.4-cm-wide and 0.12 mm high parallel-plate chamber with heparin-anticoagulated blood collected from healthy volunteers. Assuming laminar flow conditions, the shear rate at the surface of the section was 900 s^-1^, according to the formula 1.03*6 Q/(w*h^2^), where Q is the flow rate in mL s^-1^, w and h are the width and the height of the flow path in cm, respectively. This intermediate shear rate was chosen as an adequate model of the rheological situation in medium-sized arteries, where deposition of both platelets and fibrin is enabled 30. Before the perfusion, the sections were blocked with 2 w/v% bovine serum albumin (BSA) in 0.05 mol L^-1^ Tris buffer pH 7.4 containing 0.1 mol L^-1^ NaCl and 0.02 w/v% NaN_3_ (BSA-TBS) for 45 min and the 90-s perfusion was followed by a 30-s wash with 1.5 mmol L^-1^ KH_2_PO_4_, 8.1 mmol L^-1^ Na_2_HPO_4_ buffer pH 7.4 containing 137 mmol L^-1^ NaCl and 2.7 mmol L^-1^ KCl (PBS). The sections were thereafter fixed in acetone at 4 °C for 10 min and the deposited platelets and fibrin were double-stained for indirect immunofluorescence microscopy using (1) the mouse monoclonal antibody against human GpIIb/IIIa (sc-53417, Santa Cruz Biotech) followed by Goat-anti-Mouse IgG-Alexa Fluor 633 for platelets, and (2) the rabbit polyclonal antibody developed against the N-terminal part of the gamma chain, which recognizes both fibrinogen and fibrin (PA5-29734), followed by Goat-anti-Rabbit IgG-Alexa Fluor 546 for fibrin(ogen) (the latter 3 antibodies were from Invitrogen, Budapest, Hungary). Confocal images in three wavelength channels were taken from the slides with a Zeiss LSM710 confocal laser scanning microscope equipped with a 10 × 0.3 objective (Carl Zeiss, Jena, Germany) using 488 nm, 543 nm and 633 nm excitation laser lines and emissions were detected in the ranges of 500-530 nm, 565-585 nm and 650-690 nm, respectively. The fluorescent nucleic acid dye TOTO-3* (Invitrogen, Budapest, Hungary) was used to visualize cells in the tested vessels. Each vessel sample was perfused in triplicates. Depending on the section size, 5-8 different images were taken of each perfused cryosection to survey the whole cross-sectional area of the vessel. Quantification of platelet and fibrin(ogen) coverage to the vessel wall was performed with the Image J software (NIH, Bethesda, MD, USA) selecting the region of interest, calculating its surface area in pixels and setting a threshold intensity value for automatic identification of the percent area covered by platelets or fibrin(ogen).

### Vasoreactivity studies

The vasoactive function of hPSC-EC was assessed by investigating vasomotor responses of isolated rat aortic rings 9. *In vitro* organ bath experiments allowed studying the vasoactive characteristics of hPSC-EC in an angio-myograph system (*Radnoti Glass Technology*)*.* The aortic rings of young, adult Sprague-Dawley rats (250-350g, *Charles River Laboratories*) were used. Animals received general housing: room temperature and 12 h light/dark cycles, standard laboratory diet, and free access to food and water. The animals received general anesthesia with intraperitoneal injection of sodium-pentobarbital (60 mg/kg) solution. The ascending thoracic aorta was isolated and immediately immersed into 4°C Krebs solution (118 mM NaCl, 4.7 mM KCl, 1.2 mM KH_2_PO_4_, 1.2 mM MgSO_4_, 1.77 mM CaCl_2_, 25 mM NaHCO_3_, 11.4 mM glucose; pH 7.4). Fat and connective tissue debris were cut off the peri-adventitial region, and the aortic samples were cut into 4 mm rings. After preparation, the aortic rings were mounted on stainless steel hooks in individual organ baths and connected to isometric force transducers. Each glass chamber contained 30 mL of pre-warmed (37 °C) Krebs solution, aerated with 95% O_2_ and 5% CO_2_. After setting the basal tension of the vessels, the functional precontraction and the viability of the endothelium and smooth muscle cells were verified. The vasoactive function of stem cell-derived endothelial cells was tested. Experimental groups included hESC-EC, hiPSC-EC, and HUVEC supernatants. As control samples, we have used EGM-2 to avoid biasing our results with vasoactive effects of EGM-2, given that it contains vasoactive substances. Basal tension was set at 20mN of force, followed by incubation for 60 min. To reach the maximum plateau of contraction, the vessel rings were treated with KCl (80mM). Next, the rings were precontracted with phenylephrine (10^-6^ M, *Sigma-Aldrich*). The viability of the endothelium was assessed by endothelium-dependent vasodilatory response to acetylcholine (ACh) (10^-9^ - 10^-4^M, *Sigma-Aldrich*). The viability of smooth muscle cells was assessed by endothelium-independent vasodilatory response to sodium-nitroprusside (SNP, 10^-10^ - 10^-5^M, data not shown) (*Sigma-Aldrich*). To the examined vasoactive responses of hESC-EC/hiPSC-EC products, a supernatant of 10^6^ hPSC-EC (5 mL) was added to each vessel bath. Any vasodilation or vasoconstriction was registered, and data from the contraction force was digitalized and stored on LabChart7 for further analysis (*Powerlab*).

### Subcutaneous transplantation of endothelial cell constructs into athymic nude rats

To investigate *in vivo* survival, proliferation, angiogenic activity, and transcriptome changes, hESC-EC and hiPSC-EC were engrafted into three-months-old, male athymic nude rats (Crl:NIH-Foxn1rnu, Charles River, Wilmington, Massachusetts, USA). As these rats are T-cell deficient, human cellular grafts do not induce immunogenic host reactions 31. Animals were housed under pathogen-free conditions. Upon general anaesthesia, animals received ketamine and xylazine (80-100 mg/kg and 5-10 mg/kg, respectively, i.p.), as per guidelines. We injected hESC-EC (n = 8 rats, from 3 independent experiments in total), hiPSC-EC (n = 6, from 2 independent experiments), or HUVEC (n = 6, from 3 independent experiments) mixed within Matrigel extracellular matrix (BD Biosciences) subcutaneously. Injected plugs contained 10^6^ cells, Matrigel (250 µL), heparin (64 U/mL), recombinant murine basic FGF (80 ng/mL, R&D Systems, Minneapolis, Minnesota, USA), and EGM2 (70 µL, Lonza). To test endogenous cell-based angiogenesis, Matrigel mixed with growth factors and endothelial cell culture media (n = 10, from 3 independent experiments) was used as control. Angiogenesis was imaged with small animal PET/MRI and small animal SPECT/CT at two weeks of follow-up. Animals were sacrificed, and all implantation sites and plugs were analyzed. Samples were stored for histology or RNA isolation in Tri Reagent (*Sigma-Aldrich*) at -80 °C. Schematic study design for small and large animal models are shown in Figure S11.

### Imaging of vascularization using PET/MR and SPECT/CT

PET/MRI images were recorded on a nanoScan integrated PET/MRI system (Mediso, Budapest, Hungary). Dynamic PET scanning was initiated immediately after the injection of radioactive probes and continued for 120 min. The acquisition took place in 1-5 coincidence mode with 5 ns coincidence window, 400-600 keV energy window, and 94.7 mm scan range. A 3D expectation maximization (3D EM) PET reconstruction algorithm (Nucline-TeraTomo, version: 3.00.021, Mediso, Hungary) was applied to produce PET images, including corrections for attenuation and scatter, dead time, decay, and randoms. After 8 iterations the reconstruction ended, which resulted in images with 0.1 mm voxel size and time frames of 8 × 15 min. MR scanning was performed immediately after PET scans were obtained. Supporting MR images were acquired with nanoScan PET/MRI 1T magnet (Aspect, Rehovot, Israel) with a horizontal bore magnet, using a solenoid Tx/Rx coil (diameter of 35 mm), and 450 mT/m gradients. T2-weighted two dimensional and gradient echo 3D images were acquired. The images of the two modalities were automatically fused using Fusion software (Version: 3.03.089, Mediso, Hungary) and quantitatively analyzed in operator-defined 3D Volumes of Interest (VOI) with Vivoquant software (inviCRO, Boston, Massachusetts, USA). Reconstructed multi-modal animal image volumes were analyzed in the VOIs of the identified cellularized graft volumes for all radiotracers in terms of radioactivity concentration (kBq/mm^3^) and in Standardised Uptake Value (whereby the radioactivity concentration is normalized by the proportion between injected radioactivity and the weight of the animal.

To measure perfusion of implanted constructs at two weeks follow-up of transplantation, animals received ^99m^Tc-labelled human serum albumin intravenously via the tail vein (Albumon kit for radiolabeling, Medi-Radiopharma Ltd., Érd, Hungary). The integrin expression of implanted cellular plugs was monitored using a molecular imaging probe based on RGD (Arg-Gly-Asp) loop tripeptide coupled to the metal ion chelator 1,4,7-triazacyclononane-N`,N``,N``` triacetic acid (NOTA-RGD). This α_v_β_3_ integrin affine agent NOTA-RGD2 was a generous gift from Dr Cheng, Stanford University 32. The chelator moiety allowed radiolabeling the molecule with ^68^Ga isotope for PET imaging. The intensity of radioactivity from higher perfusion levels with radiolabeled albumin is used as a surrogate marker of increased blood flow. Combining SPECT modalities with CT and MRI enables the localization of areas with different extent of blood supply. Radiolabeling of NOTA-RGD2 was performed according to the published procedure as reported elsewhere 32. ^99m^Tc radiolabeling of human serum albumin in the form of a lyophilized kit formulation (Albumon kit for radiolabeling, Medi-Radiopharma Ltd., Érd, Hungary) were applied following the kit instructions for use with ^99m^Tc eluate from a GE TechneKow ^99m^Tc generator (General Electric, Maryland Heights, MO, USA). SPECT imaging was performed on the injected animals at a 10% wide gamma energy window of 140keV for ^99m^Tc. Rats were fitted into a heated animal bed (Multicell, Mediso, Hungary) at 37 °C, and they were imaged using a NanoSPECT/CT. Silver Upgrade is a dedicated small animal imaging system equipped with 1.2 mm diameter multiplexed multi-pinhole collimators in a helical scan mode. 40-min scans were acquired from the tail base to the nose of the animals. SPECT projections were reconstructed using a Tera-Tomo iterative algorithm. CT scans were performed with 65 kV voltage and reconstructed with a voxel size of 60 microns using a ray-tracing algorithm of the system. The parts of the implanted plugs having contact with neighboring tissues were defined as outside parts. An important endpoint in the rat studies for optimization assessment was lower limb perfusion post cell implantation. This allowed screening of the different cell type grafts for translatable, clinically verifiable effects of perfusion increase.

All modality (PET, MRI, SPECT, CT) image series of every animal has been cross-referenced and co-registered in the same virtual image space using VivoQuant. This allowed us to co-register and combine modalities at will during image analysis. Thus, it was possible to combine SPECT with MRI, as shown in representative figures.

### 3D immunohistochemical analysis and quantification of angiogenesis

Plugs with surrounding tissue were explanted, fixed in formaldehyde, paraffinized and prepared for immunofluorescence staining. Blocks were cut into 5 µm slices and stained with anti-human CD31 antibody (BD Biosciences #557703), or standard hematoxylin-eosin stain. Immunohistochemistry images were captured in Axio Scan Z1 microscope (Zeiss, Oberkochen, Germany) and Pannoramic 250 whole slide scanner (3Dhistech, Budapest, Hungary). Image processing and analyses were performed in 3DHisTech platform (3DHisTech, Budapest, Hungary), which enabled 3D reconstruction of whole slides in serial sections, tissue segmentation, and morphometric studies. Furthermore, the QuantCenter software was functional for cell nuclei detection, cytoplasm filtering, and merging regions of interest to directly quantify the angiogenic activity of hESC-EC, hiPSC-EC, and HUVEC.

### Engineering vascular grafts

For 3D vascular tissue engineering, endothelial cells were seeded onto 3D decellularised biomatrices for *in vivo* use. Canine aortic sections were harvested under sterile conditions from euthanized animals in earlier experimental studies and were prepared as described previously 33, 34. Aortic samples were stored in Medium 199 with Earle's salts (ThermoFisher Scientific, Waltham, MA, USA) containing 10% dimethyl sulfoxide (DMSO) at -80 °C until further use. Next, aortic samples were thawed and decellularised, as described above. Acellular aortic grafts were coated with fibronectin (0.5%, 30 min) to facilitate endothelial adhesion. Human iPSC-EC were seeded onto the luminal surface of matrix segments (100,000 endothelial cells/cm^2^ matrix) in a spinner flask bioreactor (Corning, Basel, Switzerland) under physiologic conditions (37°C, 85% humidity, 21% O_2_) in EGM2 media. The efficiency of endothelial recellularization was verified by immunohistochemistry, confocal microscopy and qRT-PCR analysis. Vascular segments were labelled with anti-CD31 antibody (1:100) and Ku80 (1:100, Abcam, Cambridge, UK) to quantify reendothelialization and verify the human specificity of seeded endothelial cells. Samples were investigated by confocal laser scanning microscopy (Zeiss LSM780).

### Large animal model of aortic replacement surgery

Decellularised aortic grafts reseeded with hiPSC-EC were implanted as aortic interposition grafts at abdominal aortic surgeries; these grafts were used to replace the infrarenal aortic segment in an end-to-end fashion. As a control arm of the study, decellularised aortic grafts without cell seeding were also tested in chronic experiments with a six-month follow-up. Beagle dogs (WOBE Kft., Budapest, Hungary) of both sexes (n = 4), weighing 15.5±0.4 kg were acclimatized for 3 weeks. On the day of the surgery, dogs were premedicated with acepromazine (0.03 mg/kg i.m.) and the cephalic vein was cannulated. The animals received cefuroxime (750 mg i.v.) for preoperative antibiotic prophylaxis, were anesthetized with pentobarbital (30 mg/kg initial bolus and then 25 mg/kg/h i.v.), ketamine (1 mg/kg initial bolus and then 1.5 mg/kg/h i.v.) and tramadol (100 mg i.v. bolus), were paralyzed with pancuronium bromide (0.1 mg/kg as a bolus and then 0.2 mg/kg/h i.v.) and were intubated endotracheally. The dogs were ventilated with a mixture of room air and O_2_ (FiO_2_ = 60%) at a frequency of 12-15/min and a tidal volume of 15 mL/kg/min. Venous blood samples were regularly collected to analyse blood gases, electrolytes, pH, and parameters of blood coagulation. Basic intravenous volume substitution was carried out via the venous cannula with Ringer's solution at a rate of 1 mL/min/kg. According to potassium, bicarbonate, and base excess values, substitution included administration of potassium chloride and sodium bicarbonate (8.4%). Vital functions were continuously monitored during surgery.

The infrarenal segment of the abdominal aorta was exposed via the retroperitoneal approach. After systemic anticoagulation with unfractionated heparin (300 U/kg i.v.) and controlling blood pressure with intravenous glyceryl trinitrate if necessary, the infrarenal aorta was cross-clamped for 39±7 min, an appropriate length of the aorta was removed and replaced with the recellularised graft by end-to-end anastomoses using 5-0 Prolene sutures. Heparin was antagonized with protamine (300 U/kg i.v.) over 10 min, and finally, the retroperitoneum was closed. Vital functions were continuously monitored in the first 4 postoperative hours, then in every 4 h during the first 12 h and then twice daily: general appearance and behavior, activity, water and food intake, excretion, diuresis, and signs of potential pain or bleeding. For postoperative analgesia tramadol (100 mg oral) and meloxicam (15 mg oral) were used daily for 5 days. Aspirin (100 mg p.o.) was administered once daily for antiplatelet effects. Postoperative antibiotics comprised of amoxicillin-clavulanic acid (500 mg p.o. twice daily) for the one-week duration. Given the xenotransplantation of human cells, immunosuppressant medication was initiated in the early perioperative phase. Immunosuppressant medication consisted of an intravenous bolus injection of corticosteroid (methylprednisolone, 125 mg) and cyclosporine A (CsA, 50 mg) during anesthesia and 10 mg oral CsA daily. Monitoring of wound healing, treatment, and hygiene was as per standard clinical care daily.

### Real-time fluorescent endothelial cell tracking *in vivo*

For *in vivo* real-time endothelial cell tracking, the Cellvizio fiberoptic endoscopic system (Mauna Kea Technologies, Paris, France) was used. The system is set up from endoscopic fiber holding laser emission lights and camera to detect signals. Cellvizio enabled real-time *in vivo* imaging of implanted grafts upon abdominal aortic surgeries of beagle dogs. Prior to implantation of tissue-engineered vascular grafts, hiPSC-EC were stained with QTracker 525 and QTracker 655 (Thermo Fisher Scientific) (by delivering fluorescent nanocrystals into the cytoplasm of live cells). Dye wavelengths avoided autofluorescence emission of the bioscaffolds: this was tested in *in vitro* preliminary experiments with fluorescence microscopy. The resolution of the Cellvizio system with an S1500 optics end piece was 3 μm, with continuous *in vivo* imaging over time using an 8 Hz frequency sampling rate. Field-of-view of the fiber optic system is 800 μm in diameter. Given that the collagen component of the decellularized extracellular matrix had strong autofluorescence, stained cells were injected directly into the matrix as procedural control. Real-time videos or still images are exportable following image acquisition. Cellvizio .tiff format images were transformed into histograms using the ImageJ (NIH, Bethesda, US) software, and the mean, standard deviation, range, skewness and kurtosis values of these histograms were calculated using Matlab (MathWorks, Natick, MA, US) in a self-developed code. Textural features skewness and kurtosis are indicators of underlying collagen fluorescent intensity, thus collagen structure redistribution. Changes in the extracellular matrix texture were quantitated as skewness and kurtosis after cell-seeded regions were excluded from regions of interest.

### Ultrasonography for patency

Implanted tissue-engineered vascular grafts were monitored by duplex ultrasound to evaluate their size and patency and visualize a possible hematoma or an inflammatory fluid collection in the surrounding tissue. Diameter (mm), and peak systolic and end-diastolic velocities (cm/s, PSV and EDV, respectively) of each graft at three points (proximal anastomosis, mid of grafts, and distal anastomosis) were obtained at 1-week follow-up.

### Large animal CT scan

We performed CT angiography on a 256-slice multidetector-row scanner, (Brilliance iCT, Philips Healthcare, Cleveland, Ohio, USA) to evaluate conduit anatomy and surface at 5 days follow up of implantation. The animals received short-acting sedation carried out via intravenous sodium pentobarbital. CT exam was initiated 15 min after reaching a stable anaesthetic plane. A 20-gauge venous cannula in the cephalic vein was connected to the injector. The standard CT protocol consisted of a non-contrast abdominal scan. Then, with the bolus-tracking technology over the same range, the arterial phase of the iodinated contrast media was acquired. CT scans of the implanted aortic graft were visualized and analyzed using Philips IntelliSpace Portal Version 6.0 (Philips Healthcare).

### Histology and immunohistochemistry

For standard histology and immunohistochemistry, vascular grafts were explanted, fixed in 4% paraformaldehyde, and embedded in paraffin. Paraffin blocks were cut into 5 µm sections and stained for hematoxylin-eosin, Masson's trichrome, and orcein to study morphology, connective tissue, and elastic tissue, respectively. The following primary antibodies were used for immunostaining: anti-Ku80 (EPR9111(B), Abcam), anti-CD31 (557703, BDBiosciences), anti-human CD31 (DAKO, clone JC70A), anti-smooth muscle actin (Abcam), and Hoechst. Alexa Fluor 488 and 568 (ThermoFisher Scientific) conjugated secondary antibodies were used. DAPI (4',6-Diamidino-2-phenylindole) nucleus staining was also used. Images were captured in Zeiss Axio Imager 2 or Nikon microscope (Eclipse 80i) connected to a camera (DS-Ri1; Nikon, Tokyo, Japan) and Elements software.

### RNA-seq

RNA integrity and quantitation were assessed using the RNA Nano 6000 Assay Kit of the Bioanalyzer 2100 system (Agilent Technologies, CA, USA). The Ribo-Zero Plus rRNA Depletion kit was used to remove abundant RNA using enzymatic depletion (Illumina, CA, USA). Sequencing ready libraries were prepared with NEBNext Ultra Directional RNA Library Prep Kit for Illumina (New England Biolabs, MA, USA) following manufacturer's recommendations and index codes were added to attribute sequences to each sample. Products were purified with AMPure XP beads (Beckman Coulter, CA, USA) and library quality was assessed on the Agilent Bioanalyzer 2100 system (Agilent Technologies, CA, USA). According to the manufacturer's instructions, the clustering of the index-coded samples was performed on a cBot Cluster Generation System using PE Cluster Kit cBot-HS (Illumina, CA, USA). After cluster generation, the libraries were sequenced on an Illumina platform, and paired-end reads were generated. Raw reads in FASTQ format were firstly processed through fastp. In this step, clean data (clean reads) were obtained by removing reads containing adapter and poly-N sequences and reads with low quality from raw data. Clean reads were aligned to the reference genome using the Spliced Transcripts Alignment to a Reference (STAR) software. FeatureCounts was used to count the read numbers mapped of each gene. And then, RPKM (Reads Per Kilobase of exon model per Million mapped reads) of each gene was calculated based on the length of the gene and reads count mapped to this gene. RPKM considers the effect of sequencing depth and gene length for the reads count at the same time. Differential expression analysis between two conditions/groups (three biological replicates per condition) was performed using DESeq2 R package. DESeq2 provides statistical routines for determining differential expression in gene expression data using a model based on the negative binomial distribution. Enrichment analysis was carried out using clusterProfiler package, including GO (Gene Ontology), DO (Human Disease Ontology), KEGG (Kyoto Encyclopedia of Genes and Genomes), Reactome and DisGeNET databases. Ingenuity Pathway Analysis platform (Qiagen) was also used for network analyses. RNAseq datasets are deposited in NCBI Sequence Read Archive (SRA, BioProject accession number No. PRJNA706134), database, https://www.ncbi.nlm.nih.gov/sra/PRJNA706134.

### Real-time PCR of implanted plugs and grafts

After two weeks of *in vivo* subcutaneous conditioning in rats, implanted plugs were re-isolated and collected into TriReagent (Merck). Total RNA was isolated using the RNeasy Mini Kit (Qiagen, Hilden, Germany). Single-stranded cDNA was generated from 500 ng total RNA by High Capacity cDNA Reverse Transcription Kit (Thermo Scientific). For large animal model, TaqMan PreAmp Master Mix Kit (ThermoFisher) was used to preamplify cDNA from isolated samples targets. CD31, EphrinB2, EphB4, Notch1, Notch2, VE-cadherin, ACTA, YAP1, and TAZ mRNA levels were assessed by TaqMan gene expression assays (CD31: Hs00169777_m1, Notch1 Hs00384907_CE, Notch2 Hs00247288_CE, EphrinB2 Hs00341124_CE, EphB4 Hs01822537_cn, VE-Cadherin Hs00170986_m1, ACTA Cf02668770_m1, (dog), FSP1 Hs00243202_m1 (human), YAP1 Hs00902712_g1, and TAZ Hs00794094_m1). GAPDH (Hs02758991_g1, human or Cf04419463_gH, dog) endogenous control was used as housekeeping control. The PCR was performed on Rotor-Gene 3000 (Qiagen, Corbett Research) and StepOnePlus real-time PCR instruments (ThermoFisher Scientific) and the relative expression was determined by the ΔΔCt method in which fold increase = 2^-ΔΔCt^. Functional association network analyses were additionally confirmed by Ingenuity Pathway Analysis software (Qiagen).

### Statistical analysis

Statistical analysis was carried out using GraphPad Prism 9 software (San Diego, Ca, USA). Data are presented as the mean ± standard error of mean (SEM). Statistical analyses were performed by Student's t-test, Wilcoxon matched-pairs signed-rank test, Kruskal-Wallis non-parametric test, two-way ANOVA or one-way ANOVA with Tukey or Dunnett post hoc tests. *p* values less than 0.05 were considered statistically significant. Statistical differences in the coverage of the separate vessel wall layers (intima, media, adventitia) were analysed with a two-sample Kolmogorov-Smirnov distribution test at a threshold of significance p<0.05 with the Statistics and Machine Learning Toolbox 12.0 of Matlab 9.9 (Mathworks, Natick, MA, USA). Proteomic statistics used z score method and calculated each data point as a standard deviation from mean data in a dataset showing the normal population. For GO (Gene Ontology) enrichment analysis of vessel and Matrigel proteins, false discovery rates (FDR) < 0.05 are presented for each subcategory.

Reagents are detailed in Table S1. Further information and requests for resources and reagents should be directed to and will be fulfilled by the corresponding author, Gabor Foldes (foldes.gabor@med.semmelweis-univ.hu).

## Supplementary Material

Supplementary figures, table, and video legend.

Supplementary video.

## Abbreviations

EndoMT: endothelial-mesenchymal transition
hESC: human embryonic stem cells
hiPSC: human induced pluripotent stem cells
HUVEC: human umbilical vein endothelial cells
PET: positron emission tomography
SPECT: Single Photon Emission Computed Tomography
SUV: standardized uptake values
VEGF: vascular endothelial growth factor
