# Supplementary material for: 3D culturing of human pluripotent stem cells-derived endothelial cells for vascular regeneration
Source: Theranostics. 2022 Jun 6;12(10):4684–702. doi: 10.7150/thno.69938 (PMC9254250; doi:10.7150/thno.69938)
Supplement: Supplementary file 1 — Supplementary figures, table, and video legend. [file thnov12p4684s1.pdf]

3D culturing of human pluripotent stem cells-derived endothelial cells for vascular regeneration

Edit Gara, Eleonora Zucchelli, Annamária Nemes, Zoltán Jakus, Kitti Ajtay, Éva Kemecsei, Gábor Kiszler, Nikolett Hegedűs, Krisztián Szigeti, Iván Földes, Kristóf Árvai, János Kósa, Kraszimir Kolev, Erzsébet Komorowicz, Parasuraman Padmanabhan, Pál Maurovich-Horvat, Edit Dósa, György Várady, Miklós Pólos, István Hartyánszky, Sian E. Harding, Béla Merkely, Domokos Máthé, Gábor Szabó, Tamás Radovits, Gábor Földes

Supplementary figures 1-11

Supplementary table 1

Supplementary video

Supplementary video 1. Human hESC-derived endothelial cell plugs after subcutaneous implantation. 3DHistech imaging showing subcutaneous functional vessels with red blood cells are shown. Matrigel plug is shown blue, $CD31^+$ endothelial vascular structures, as well as red blood cells in functional vessels, are in green.

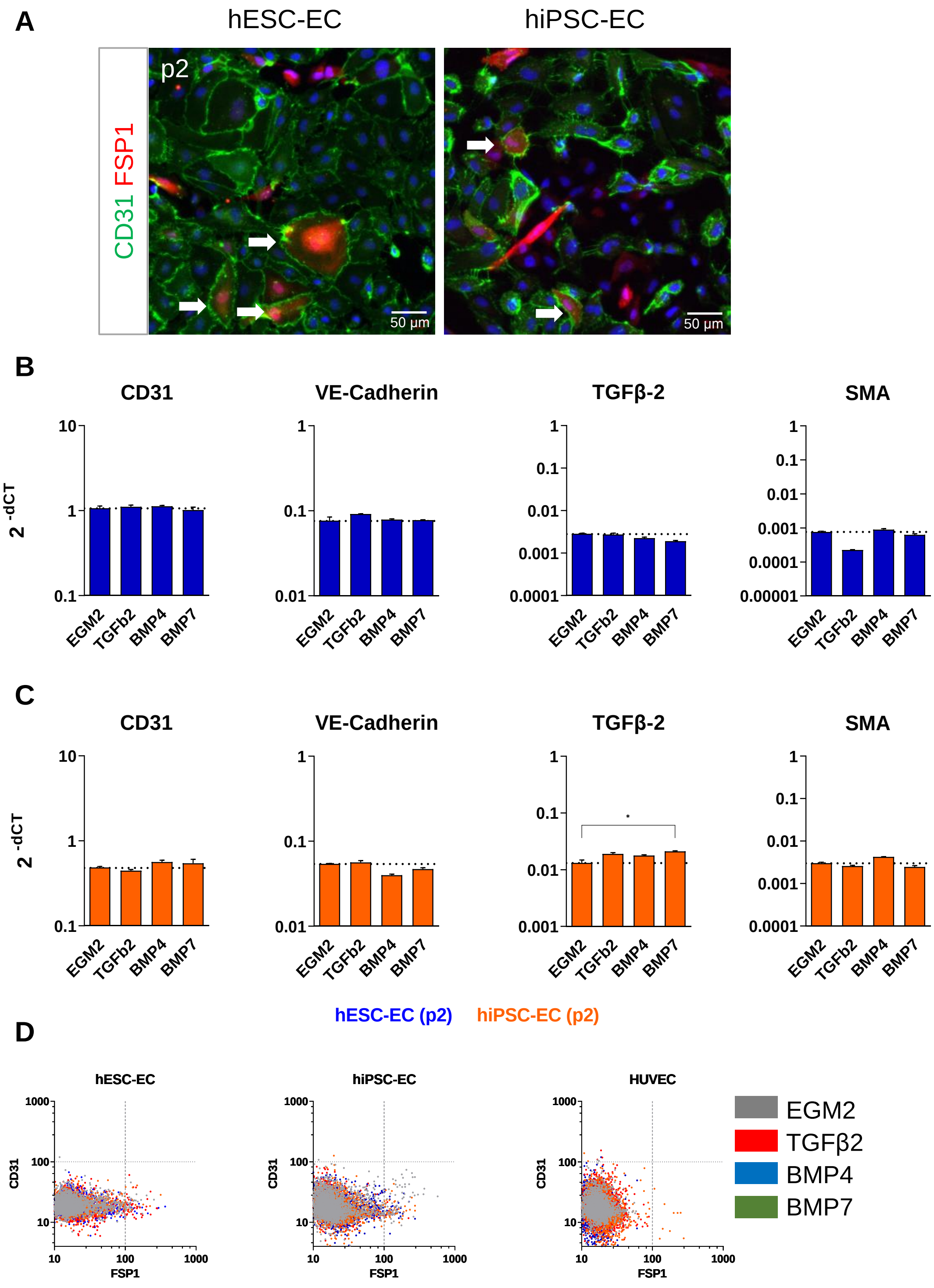

Supplementary Figure 1. (**A**) Representative immunocytochemistry images of differentiated hESC-EC and hiPSC-EC after passage 2 in culture. Single CD31+ endothelial cells (green), single FSP1+ (red) and CD31+/FSP+ cells undergoing EndoMT are shown (see white arrows). Modulation of non-endothelial drift in early passage cultures by TGFβ2. CD31, VE-Cadherin, TGFβ2 or SMA mRNA levels in passage 1 cultured hESC-EC (**B**) and hiPSC-EC (**C**) in response to TGF signalling molecules TGFβ2, BMP4, BMP7 (all 10 ng/mL), SB431542 (10 µM). Target gene expression was normalised to GAPDH expression. *p = 0.04, Kruskal-Wallis non-parametric test. (**D**) Dot plot showing population of $CD31^{+}/FSP1^{+}$ cells in response to TGFβ2, BMP4, BMP7, SB431542. Data shown on log scale, n = 3.

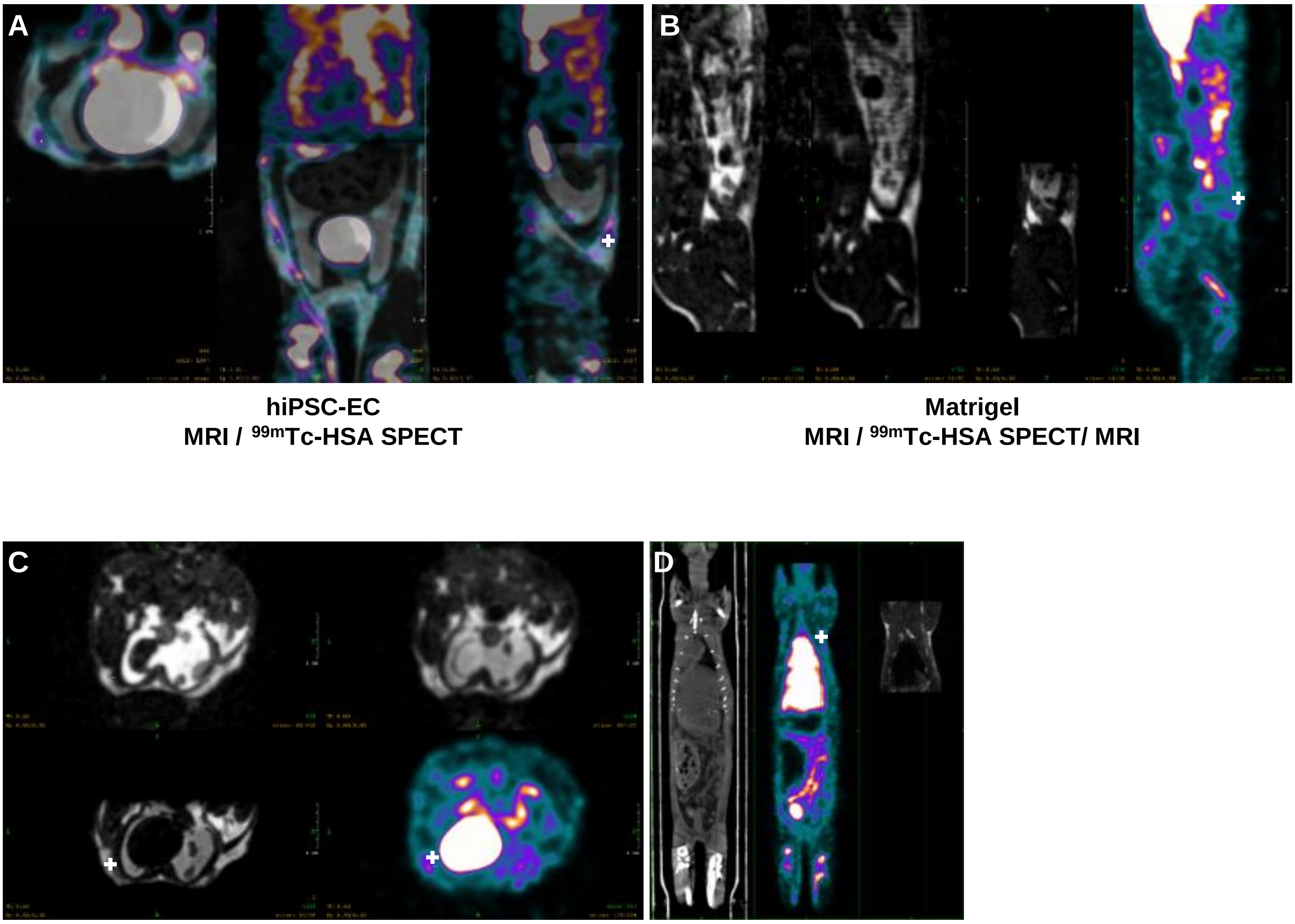

hiPSC-EC
MRI / ^{99m}Tc-HSA SPECT

Matrigel
MRI / ^{99m}Tc-HSA SPECT/ MRI

hESC-EC
MRI / ^{99m}Tc-HSA SPECT

HUVEC
CT / ^{99m}Tc-HSA SPECT/ MRI

Supplementary Figure 2. In vivo MRI/SPECT imaging shows increased perfusion after hPSC-EC implantation. MRI and SPECT modalities were combined to measure perfusion after hydrogel-based endothelial plugs were implanted. SPECT and SPECT/MRI images feature white crosshairs at the plug implantation and increased perfusion sites. MRI modality alone is also shown the same for ease of anatomical assessment. Subcutaneous blood perfusion was measured by ^{99m}Tc-human serum albumin. (b) Comparison of endothelial implants on ^{99m}Tc-human serum albumin to assess perfusion of newly formed vessels in hiPSC-EC, representative of n = 6 (**A**), human cell-free Matrigel, n = 10 (**B**), hESC-EC, n = 8 (**C**) and HUVEC plugs, n = 6 (**D**).

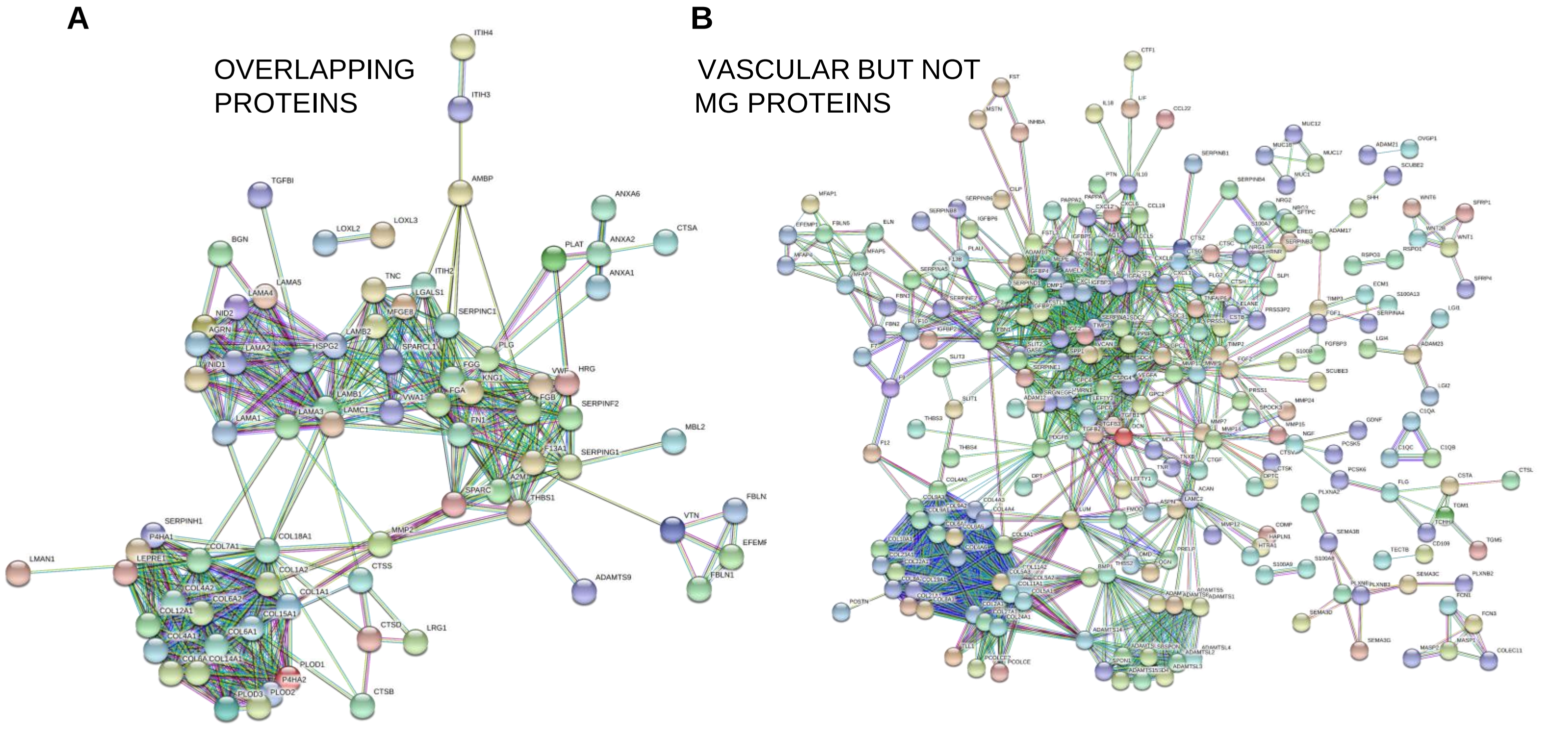

C

Biological Process (GO)		observed gene count	background gene count	false discovery rate
GO:0030198	extracellular matrix organization	52	296	1.77e-63
GO:0007155	cell adhesion	34	843	1.68e-19
GO:0030155	regulation of cell adhesion	29	623	6.08e-18
GO:0002576	platelet degranulation	16	129	3.49e-15
GO:0016192	vesicle-mediated transport	39	1699	5.88e-15
**Cellular Component (GO)**				
GO:0005576	extracellular region	80	2505	7.73e-54
GO:0031012	extracellular matrix	42	283	5.89e-48
GO:0044421	extracellular region part	63	1375	4.96e-47
GO:0062023	collagen-containing extracellular matrix	34	144	2.31e-44
GO:0005604	basement membrane	27	91	4.50e-37
**Molecular Function (GO)**				
GO:0005201	extracellular matrix structural constituent	16	73	6.95e-19
GO:0050840	extracellular matrix binding	14	51	7.94e-18
GO:0005518	collagen binding	13	61	2.12e-15
GO:0004867	serine-type endopeptidase inhibition	14	94	7.72e-15
GO:0061134	peptidase regulator activity	17	211	3.48e-14
**INTERPRO Protein Domains**				
IPR000742	EGF-like domain	20	225	4.21e-17
IPR008160	Collagen triple helix repeat	15	76	4.21e-17
IPR001791	Laminin G domain	12	58	4.14e-14
IPR013320	Concanavalin A-like lectin domain	17	220	6.60e-14
IPR002035	von Willebrand factor, type A	12	76	4.47e-13
**KEGG Pathways**				
hsa04512	ECM-receptor interaction	22	81	4.50e-29
hsa04510	Focal adhesion	20	197	6.16e-19
hsa04610	Complement and coagulation cascades	14	78	2.33e-16
hsa05165	Human papillomavirus infection	20	317	1.84e-15
hsa04151	PI3K-Akt signaling pathway	20	348	8.04e-15
**UniProt keywords**				
KW-0964	Secreted	79	1813	3.92e-63
KW-0272	Extracellular matrix	44	263	7.70e-53
KW-0732	Signal	81	3237	1.84e-47
KW-1015	Disulfide bond	74	3276	1.03e-37
KW-0325	Glycoprotein	80	4353	1.81e-36
**PFAM Protein Domains**				
PF01391	Collagen triple helix repeat (20 copies)	15	76	2.24e-17
PF00092	von Willebrand factor type A domain	12	58	3.31e-14
PF13519	von Willebrand factor type A domain	12	58	3.31e-14
PF00053	Laminin EGF domain	10	31	1.07e-13
PF00055	Laminin N-terminal (Domain VI)	7	16	2.37e-10

Supplementary Figure 3. Matrisome-based comparison of proteins in the vessel wall and Matrigel, with new matrisome subcategories between Matrigel and vascular wall tissue (database used: https://doi.org/10.1016/j.biomaterials.2019.119673). Proteins in Matrigel and vessels wall (**A**) or exclusively expressed in vessel but not in Matrigel (**B**) are shown. (**C**) Table of enrichment analysis of GO terms for proteins identified for extracellular matrix organisation, platelet regulation, and collagen-deposition. For GO enrichment analysis, false discovery rates (FDR) < 0.05 are presented for each subcategory.

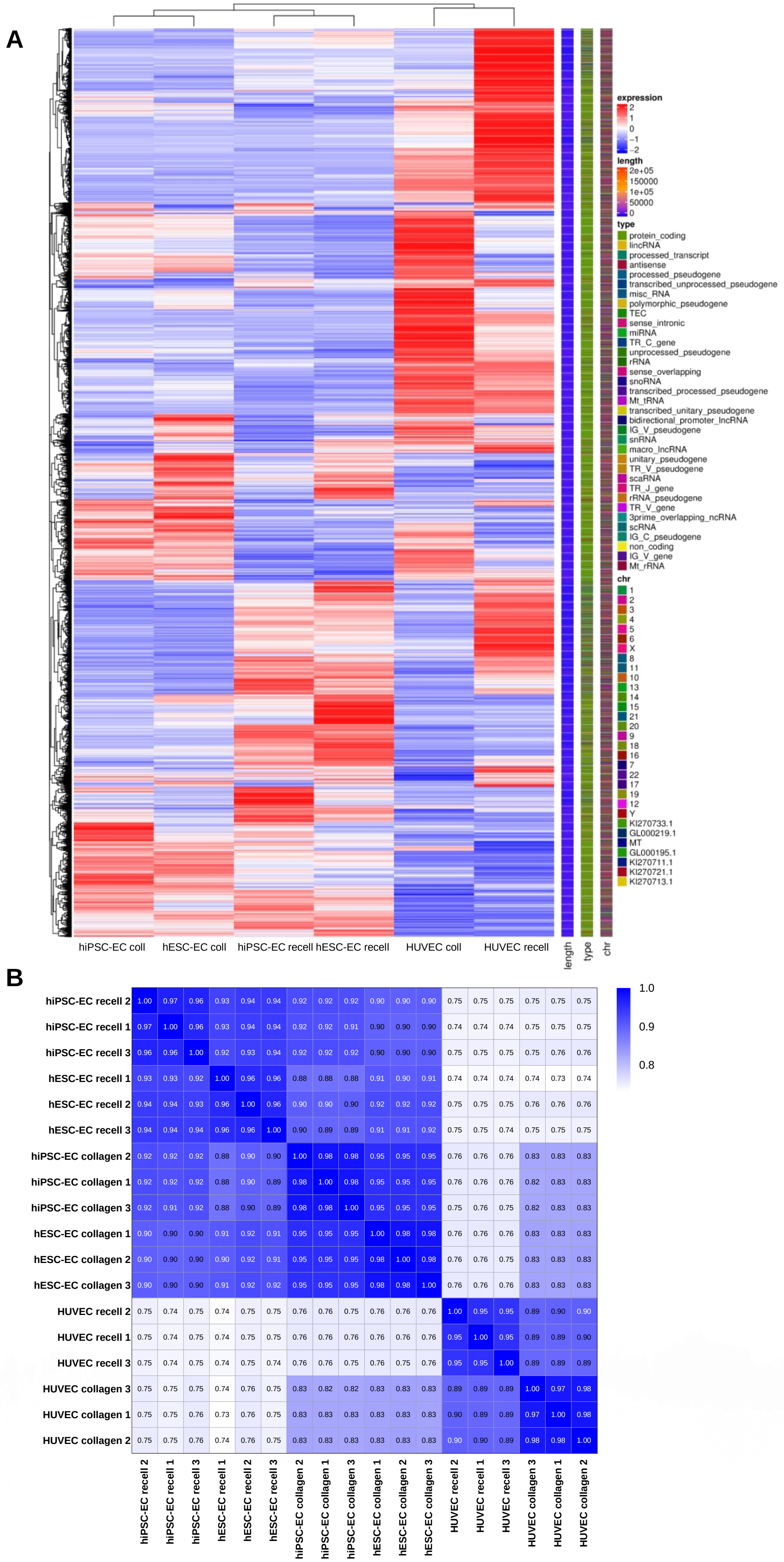

Supplementary Figure 4. Sample correlations. Global analysis of genes differentially expressed between hESC-EC, hiPSC-EC and HUVEC populations. Differentially expressed genes were identified in all pairwise comparisons between the recovered populations from our RNA-seq analysis. Hierarchical clustering (**A**) and pairwise sample Pearson correlation (**B**) were performed on genes and clusters with high expression in specific populations. Correlations are clustered by both row and column, with sample classes highlighted across the top of the heatmap. Pairwise correlation coefficients between all samples are shown.

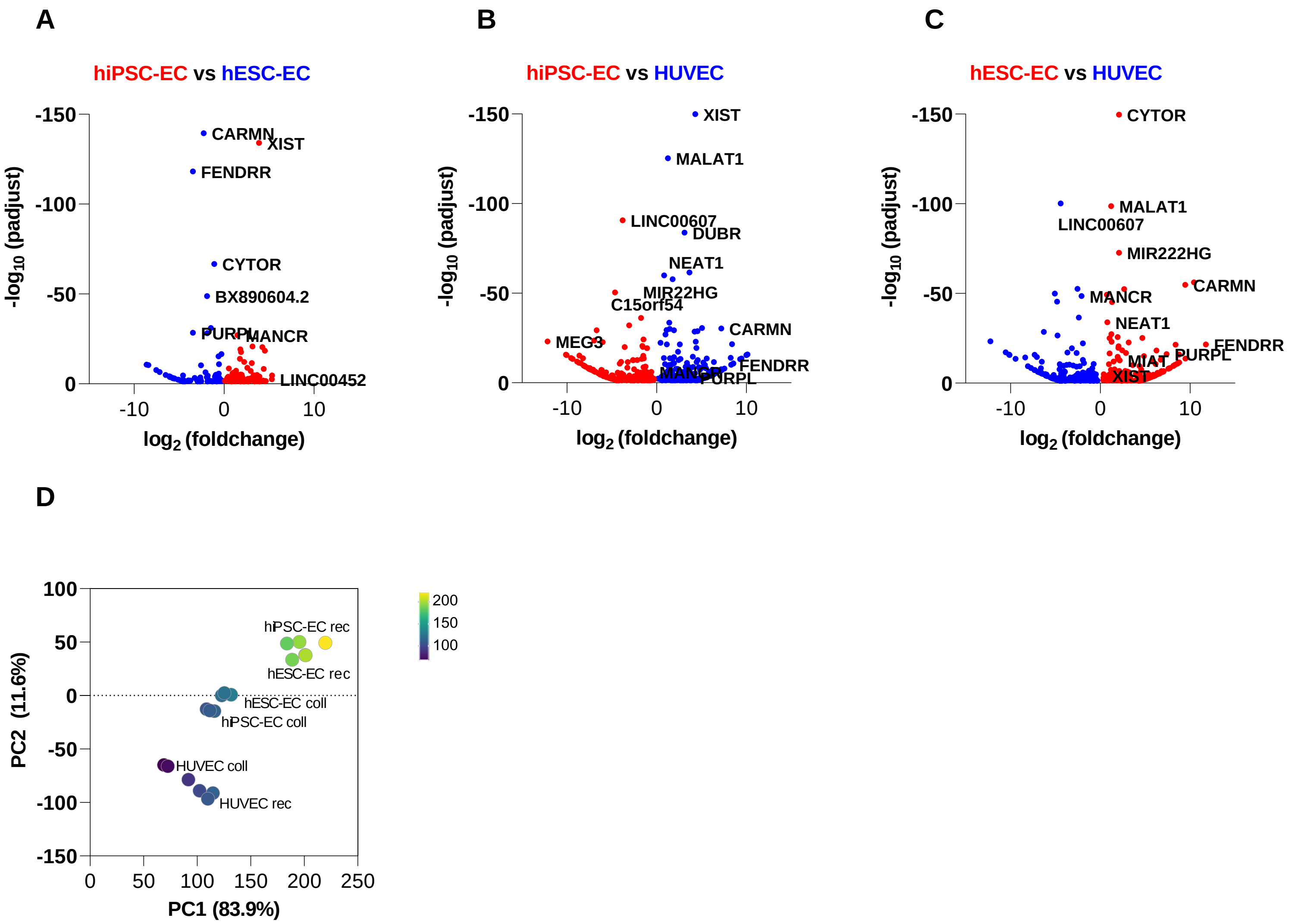

Supplementary Figure 5. RNAseq-based profiling of non-coding RNA in endothelial cells cultured on collagen or vascular matrix. Volcano plots showing the differential expression profile of H7 hESC-EC, IMR hiPSC-EC and HUVEC cultured on collagen (**A-C**). x-axis represents the $\log_2$ of the fold changes, y-axis represents the negative $\log_{10}$ of adjusted P values. Principal component analysis (**D**) show expressions of genes in the three cell types. The first two principal components of the gene expression dataset are plotted here for each of the samples. n = 3 biological replicates.

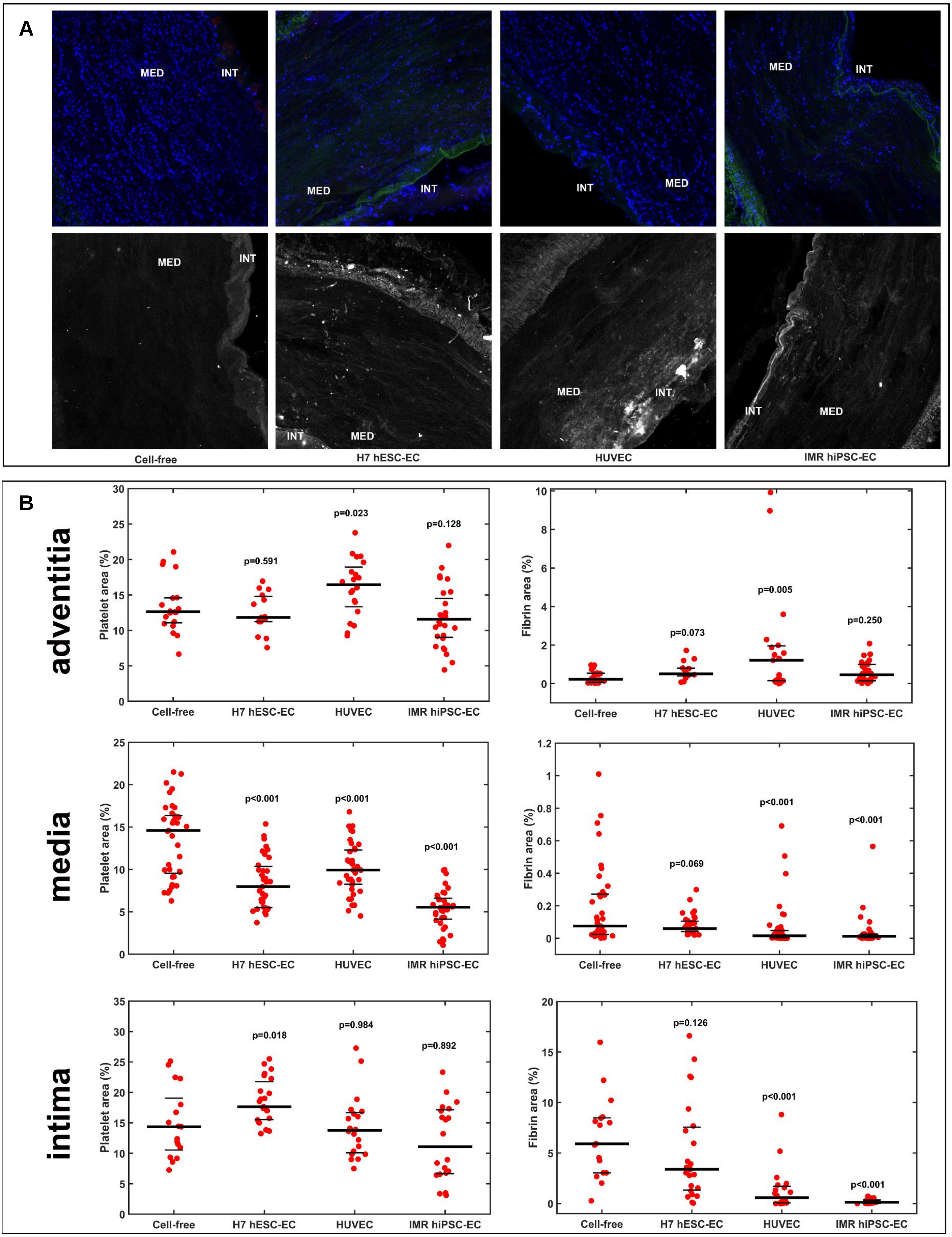

Supplementary Figure 6. Reseeding of the decellularized vessel wall dampens the deposition of platelets and fibrin(ogen) from flowing blood. Cryosections of decellularized, as well as reseeded vessel wall segments with the indicated endothelial cells were perfused with heparinized whole blood followed by indirect immunofluorescent visualization of adhered platelets and fibrin(ogen). (A) Representative images illustrate the autofluorescence for orientation (green) and the coverage of the intima (INT) and media (MED) layers with platelets (blue) and fibrin(ogen) (red), as well as the re-appearance of cell patches upon reseeding, as detected with the fluorescent nucleic acid-binding dye, TOTO-3* (black-and-white images). Images are of original magnification, displaying an 850 μm × 850 μm real square area of the vessel wall. (B) Surface coverage with platelets or fibrin(ogen) in the separate vessel wall layers. The relative area in the separate regions of interest occupied by the respective antigen (a) was quantified in 18-24 images for each layer. The indicated p-values refer to comparisons with the cell-free samples (two-sample Kolmogorov-Smirnov distribution test). Note that the red signal is barely visible in composite images due to its low level after decellularization. The panels show the medians, top and bottom quartile of the bootstrap simulated samples for each group of two samples as black lines. Differences of all pairs of medians are statistically significant at $p<0.05$.

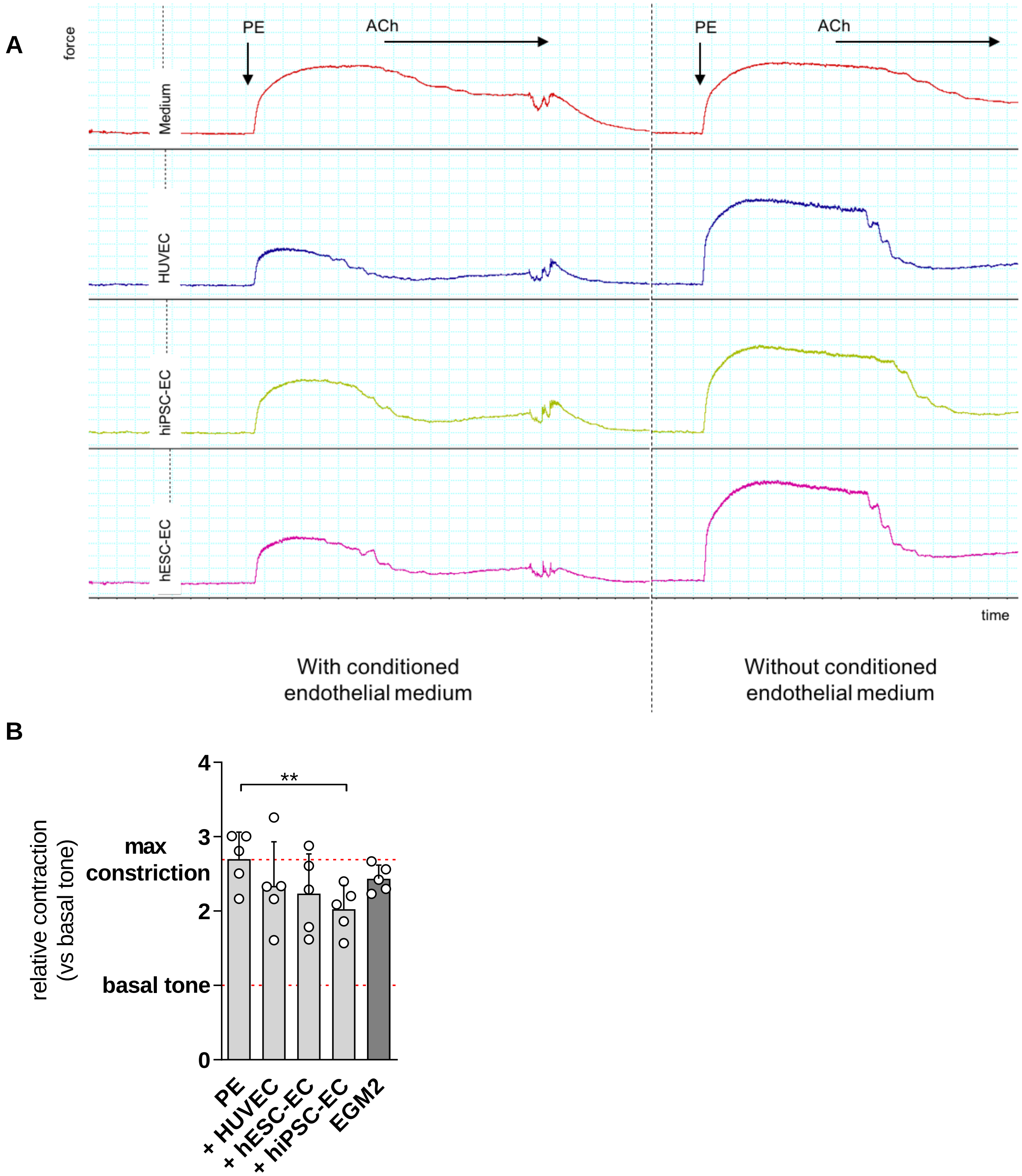

Supplementary Figure 7. Human PSC-EC show vasoactive activities in 3D culture. Vasoactive effects of human embryonic stem cell-derived endothelial cells (hESC-EC), human induced pluripotent stem cell-derived endothelial cells (hiPSC-EC), human umbilical vein endothelial cells (HUVEC) were tested in vitro in isolated vessel water-bath system. (**A**) Original traces show vasoactive effects of hESC-EC, hiPSC-EC and HUVEC supernatant on isolated rat aortic rings. Changes reflect force changes in vessel wall, positive changes on y-axis means increased force and vasoconstriction. (**B**) Bar diagram shows vasoactive effects of conditioned medium from hESC-EC, hiPSC-EC, and HUVECs on phenylephrine-induced vascular tone. Changes in vascular tone are compared to mechanically set basal tone; fold changes are presented. Phenylephrine (PE) bar shows data from recordings in Krebs solution. Data are presented as mean ± SEM. N = 11 animals / n = 44 aortic rings at each experimental group, **$p < 0.01$, one-way ANOVA with Dunnett post-hoc test.

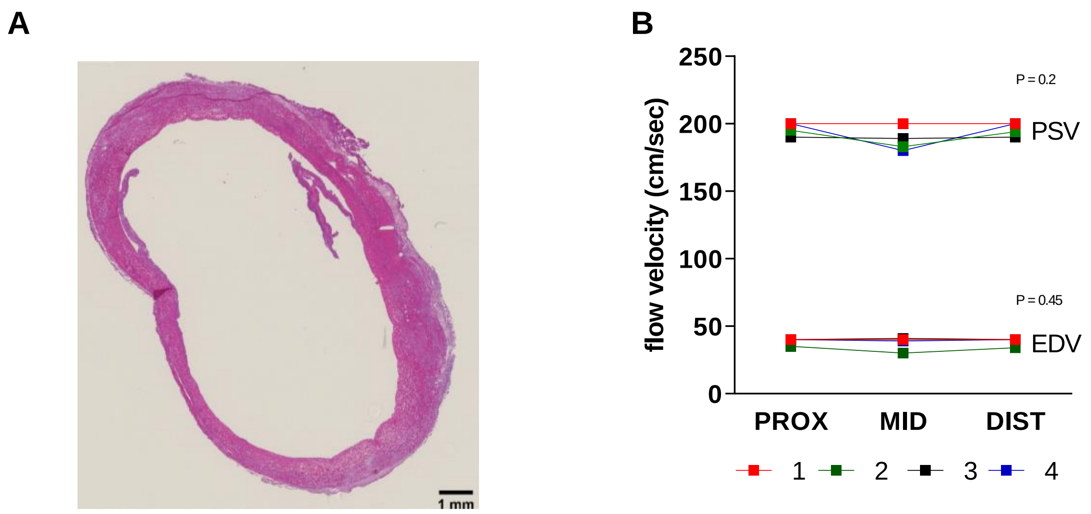

Figure 8. Graft patency. (**A**) Representative cross section H&E image of the patent vessel after 1-week follow-up. (**B**) Duplex ultrasonography shows stable patency. Peak systolic and end-diastolic velocities (cm/s, PSV and EDV, respectively) of each graft at three points (proximal anastomosis, PROX; mid of grafts, MID; and distal anastomosis, DIST) were obtained at 1-week follow-up, n = 4, two-way ANOVA.

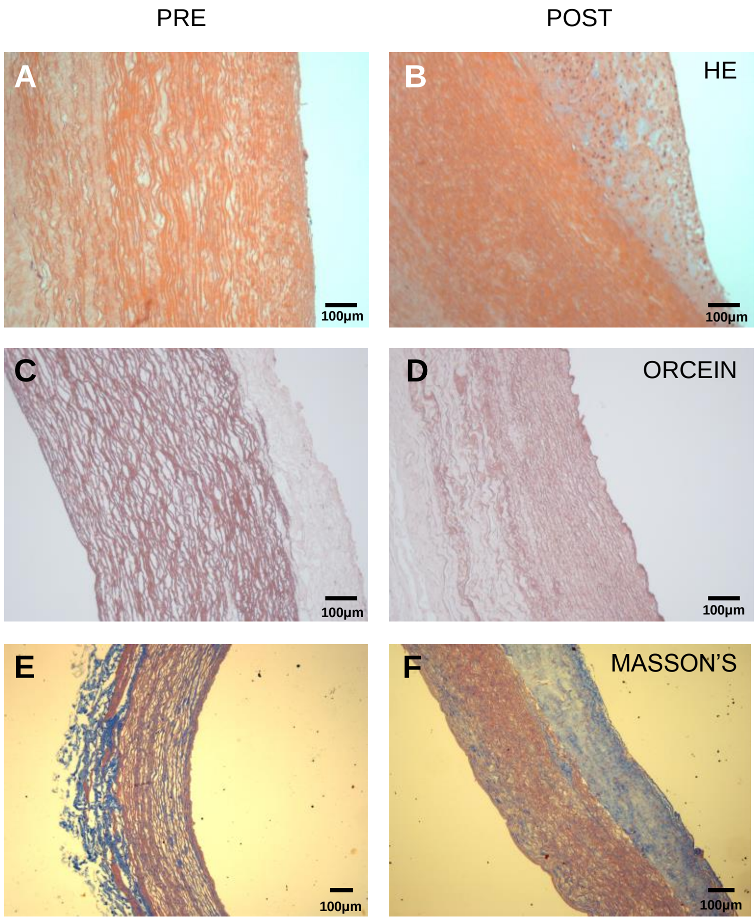

Supplementary Figure 9. Histology of decellularised aortic graft implantation in canine model at 6 months time point. Decellularised graft implantation into the peripheral artery by performing end-to-end anastomosis in dog infrarenal aorta, n = 4. Hematoxylin-eosin (**A-B**), orcein (**C-D**) and Masson's staining (**E-F**) before surgery (PRE) and at one-week follow-up (POST).

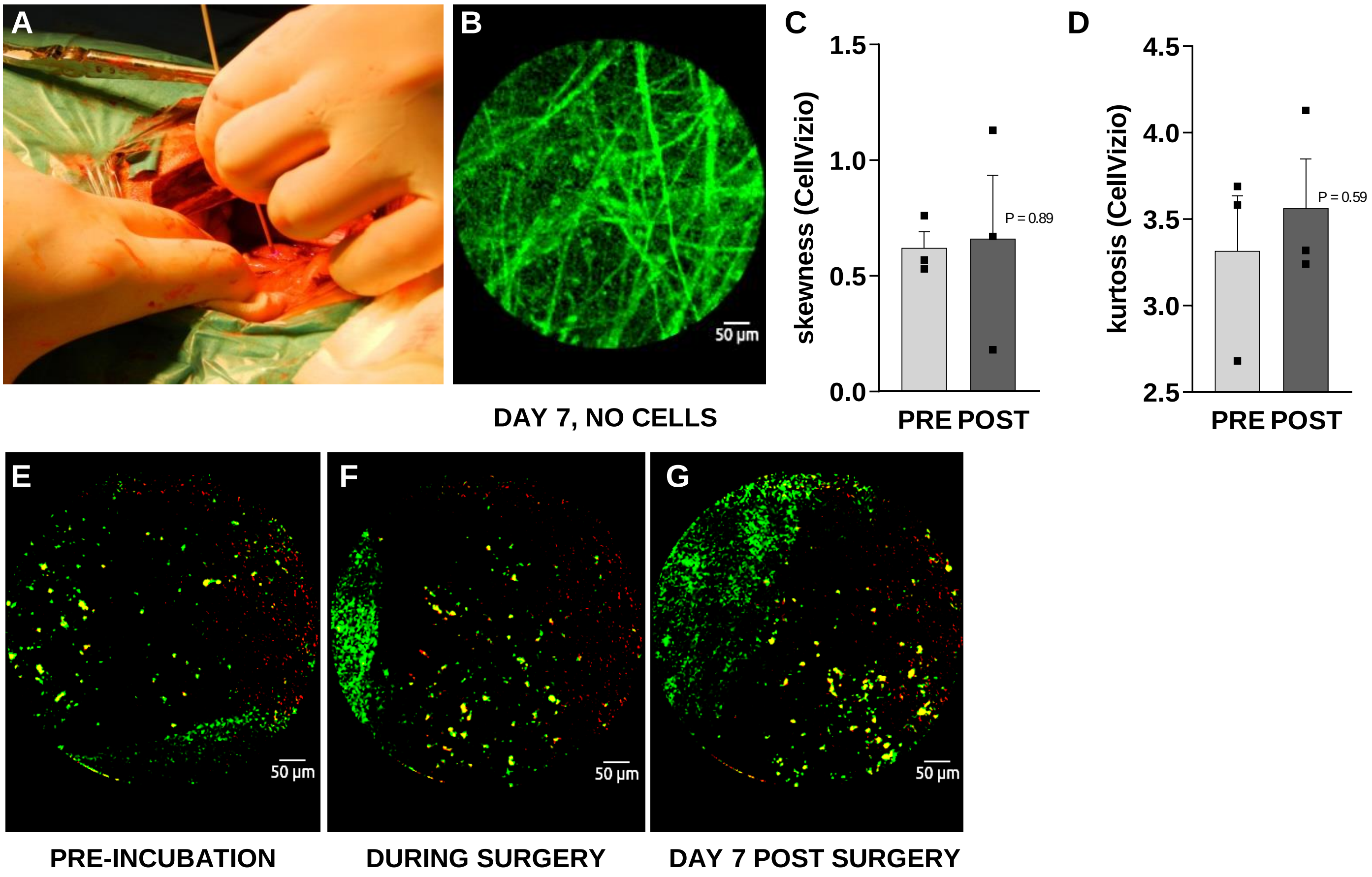

Supplementary Figure 10. Procedural control of hiPSC-derived endothelial cells by confocal fiberoptic endomicroscopy and in vivo assessment of matrices in vessel wall. (**A**) Continuous in vivo imaging over time using an 8 Hz frequency sampling rate during surgery. Still images are exported from the videos. (**B**) Representative images show decellularised vessel wall without cells on day 7. The field-of-view is 800 µm in diameter, the resolution with an S1500 optics endpiece is 3 µm. Assessment of changes in extracellular matrix morphology [texture features expressed as skewness (**C**) and kurtosis (**D**), n = 4, unpaired Student's t-test]. Procedural control for in vivo fluorescent tracking shown before (**E**), during operation (**F**), and at day 7 (**g**) after fluorescently labelled cells injected directly into the subendothelial layer of the aortic lumen. QTracker green and red vital dyes are used to improve cell signal to background and non-specific autofluorescence ratio. In all cases, a "blank scaffold" image, an only-cell containing "positive control" image and an image of directly injected "intra-aortic" cells were acquired to establish valid cellular detection images in vivo in the dog aorta. 1

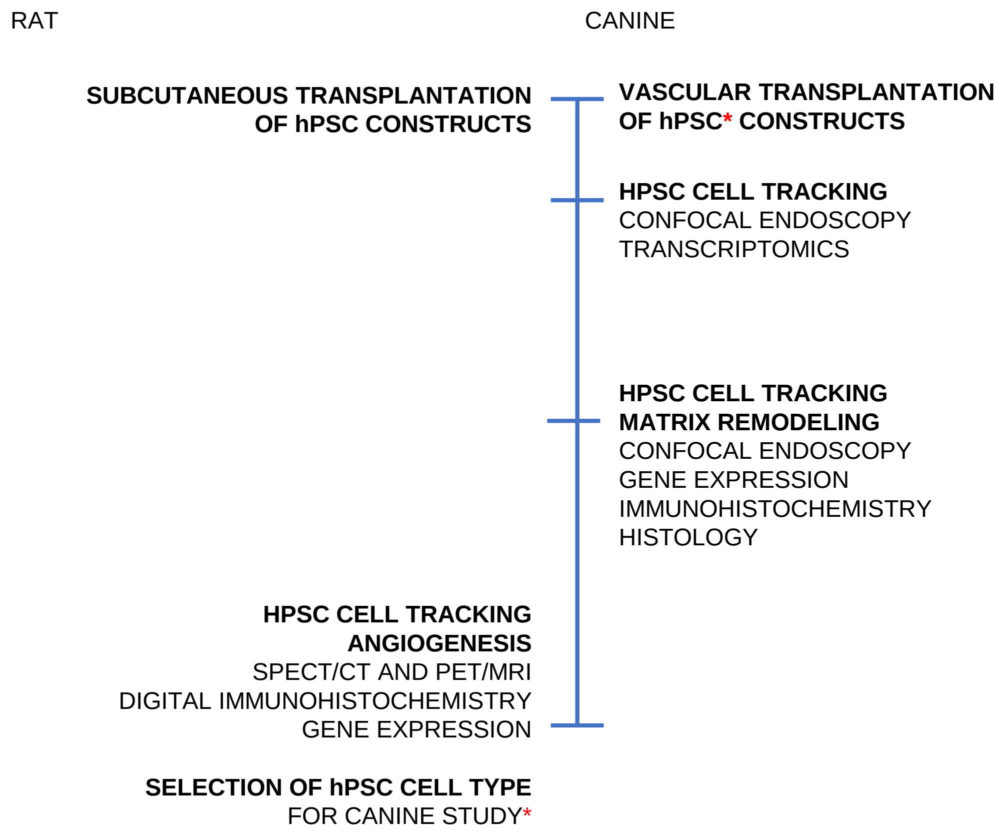

Supplementary Figure 11. Study design. Follow-up after implantation into nude rats and as aortic anastomosis in dogs. We planned a preclinical phase of cell selection (*) and optimisation in rats as a run-up for the large animal preclinical study assessing the patency and effect of those selected cells.

Supplementary Table 1. Reagents

	SOURCE	IDENTIFIER
Antibodies		
anti-CD31	Abcam	#24590/28364
anti-hu nuclei (clone 235-1)	Merck	#MAB1281
anti-human CD31	BD Biosciences	#557703
anti-human CD31 (clone JC70A)	DAKO	#M0823
anti-human Von Willebrand Factor	DAKO	#A0082
anti-Ki67 [SP6]	Abcam	#AB16667
anti-Ku80	Abcam	#AB97433
anti-mouse or anti-rabbit Alexa Fluor 546 / 488 / 568	Thermo Fischer	#A10040
anti-smooth muscle actin (rabbit polyclocal)	Abcam	#AB5694
DAPI	Thermo Fischer	#D21490
QTracker 525 / QTracker 655	Thermo Fischer	#Q25021MP
Biological samples		
Vascular tissue	Semmelweis Biobank	7891/2012/EKU
Chemicals, peptides, and recombinant proteins		
1,4,7-triazacyclononane-N`,N``,N``` triacetic acid	Stanford University	
acetylcholine	Sigma-Aldrich	# A2661
cefuroxime	Sandoz	#0055268-75-2
cyclosporine A	Elanco US	#02427885
dimethyl sulfoxide (DMSO)	Sigma-Aldrich	#67-68-5
endothelial growth medium-2 (EGM2)	Lonza	#CC-3162
gelatin	Sigma-Aldrich	#G1393
heparin	Sigma-Aldrich	#9041-08-1
ketamine	Putney	#045-290
Matrigel	BD Biosciences	#356230
Medium 199 with Earle’s salts	Thermo Fisher	#11150059
meloxicam	Boehringer Ingelheim	#141-213
methylprednisolone	Zoetis	#011-403
mTeSR1 medium	ThermoFisher	#05850
pancuronium bromide	AstraZeneca	#0015500-66-0
penicillin/streptomycin	Sigma-Aldrich	#P4333
phenylephrine	Sigma-Aldrich	# BP284
recombinant murine basic FGF	R&D System	#LS-G4842
sodium-azide	Sigma-Aldrich	#S2002
sodium-dodecyl-sulphate	Sigma-Aldrich	#436143
sodium-nitroprusside	Sigma-Aldrich	#228710
sodium-pentobarbital	Bioveta	
tramadol	Janssen	Ultram
Tri Reagent	Sigma-Aldrich	#T9424
Triton X-100	Sigma-Aldrich	# X100
xylazine	Bayer	#1470
Critical commercial assays		
Proteome Profiler Human Angiogenesis Array Kit	R&D System	#ARY007
Human Soluble Receptor Hematopoietic Array Kit	R&D System	#ARY011
Albumon kit for radiolabeling	Medi-Radiopharma	
High Capacity cDNA Reverse Transcription Kit	Thermo Fisher	#4368814
RNeasy Mini Kit	Qiagen	#74106
Cell lines		

human ESC line H7	WiCell Bank	#WA007
human iPSC line IMR-90-4	WiCell Bank	#WB65316
human umbilical vein endothelial cells (HUVEC)	Lonza	#C2519A
human aortic smooth muscle cells (AoSMC)	Lonza	#CC-2571
hu. cardiac microvascular endothelial cells (HMVEC-c)	Lonza	#CC-7030
Organisms/strains		
Rats: Sprague-Dawley rats	Charles River	
Rats: athymic nude	Charles River	Crl:NIH-Foxn1rnu
Dogs: Beagle	WOBE Ltd	
Oligonucleotides / TaqMan assays		
CD31	Thermo Fisher	Hs00169777_m1
Notch1	Thermo Fisher	Hs00384907_CE
EphB4	Thermo Fisher	Hs01822537_cn
Notch2	Thermo Fisher	Hs00247288_CE
FSP1 (S100A4)	Thermo Fisher	Hs00243202_m1
GAPDH	Thermo Fisher	Cf04419463_gH
GAPDH	Thermo Fisher	Hs02758991_g1
TAZ	Thermo Fisher	Hs00794094_m1
YAP1	Thermo Fisher	Hs00902712_g1
ACTA	Thermo Fisher	Cf02668770_m1
EphrinB2	Thermo Fisher	Hs00341124_CE
VE-Cadherin	Thermo Fisher	Hs00170986_m1
Software and algorithms		
GraphPad Prism 7	www.graphpad.com	RRID: SCR_002798
ImageJ	imagej.nih.gov/ij/	Windows/64bit mode
Fusion	Mediso	Version: 3.03.089
LabChart	Powerlab	Version: 7
Vivoquant	inviCRO	Version: 3.5
Matlab	MathWorks	uk.mathworks.com/
NIS-Elements	Nikon	Version: BR
Philips IntelliSpace Portal	Philips Healthcare	Version 6.0
